# Empagliflozin in Acute Myocardial Infarction Reduces No-Reflow and Preserves Cardiac Function by Preventing Endothelial Damage

**DOI:** 10.1016/j.jacbts.2024.08.003

**Published:** 2024-08-30

**Authors:** Panagiota Efstathia Nikolaou, Lara S.F. Konijnenberg, Ioannis V. Kostopoulos, Marios Miliotis, Nikolaos Mylonas, Anastasios Georgoulis, George Pavlidis, Carolien T.A. Kuster, Vince P.A. van Reijmersdal, Tom T.J. Luiken, Anna Agapaki, Rona Roverts, Nikolaos Orologas, Dimitris Grigoriadis, Gaëtan Pallot, Pierre Boucher, Nikolaos Kostomitsopoulos, Michael Paul Pieper, Stéphane Germain, Yannis Loukas, Yannis Dotsikas, Ignatios Ikonomidis, Artemis G. Hatzigeorgiou, Ourania Tsitsilonis, Coert J. Zuurbier, Robin Nijveldt, Niels van Royen, Ioanna Andreadou

**Affiliations:** a Laboratory of Pharmacology, Faculty of Pharmacy, National and Kapodistrian University of Athens, Athens, Greece; b Department of Cardiology, Radboud University Medical Center, Nijmegen, the Netherlands; c Section of Animal and Human Physiology, Department of Biology, National and Kapodistrian University of Athens, Athens, Greece; d DIANA-Lab, Department of Computer Science and Biomedical Informatics, University of Thessaly, Lamia, Greece; e Medical School, National and Kapodistrian University of Athens, Athens, Greece; f Department of Medical BioSciences, Radboud University Medical Center, Nijmegen, the Netherlands; g Center of Basic Research, Biomedical Research Foundation, Academy of Athens, Athens, Greece; h Electron Microscopy Center, Radboud UMC Technology Center, Radboud Institute of Molecular Life Sciences, Radboud University Medical Center, Nijmegen, the Netherlands; i Center for Interdisciplinary Research in Biology, College de France, CNRS, INSERM, PSL Research University, Paris, France; j Laboratory Animal Facilities, Center of Clinical, Experimental Surgery and Translational Research, Biomedical Research Foundation of the Academy of Athens, Athens, Greece; k Boehringer Ingelheim Pharma & Co., Mainz, Germany; l Laboratory of Pharmaceutical Analysis, Department of Pharmacy, National and Kapodistrian University of Athens, Athens, Greece; m Laboratory of Experimental Intensive Care and Anesthesiology, Department of Anesthesiology, Amsterdam Cardiovascular Sciences, Amsterdam UMC, Amsterdam, the Netherlands

**Keywords:** acute myocardial infarction, cardiac magnetic resonance, empagliflozin, microvascular injury, no-reflow

## Abstract

Empagliflozin treatment before acute myocardial infarction mainly targets the endothelial cell transcriptome. Empagliflozin treatment before and after myocardial infarction decreased no reflow and microvascular injury, leading to reduced infiltration of inflammatory cells, reduced infarct size, and improved cardiac function in mice. In diabetic patients receiving empagliflozin after myocardial infarction, perfused boundary region, flow-mediated dilation, and global longitudinal strain were improved.

## Highlights

• Empagliflozin pretreatment reverses the deregulated transcriptome of endothelial cells 2 hours after reperfusion in a mouse model of acute myocardial infarction.
• Empagliflozin treatment after acute myocardial infarction has cardioprotective potential similar to pretreatment, which is indicated by the reduced no-reflow phenomenon and infarct size and the preservation of myocardial function.
• Besides the preservation of endothelium integrity, empagliflozin treatment leads to reduced neutrophil and inflammatory monocyte infiltration in the heart and attenuates matrix metalloproteinase-2 and intercellular adhesion molecule-1 protein levels 48 hours after reperfusion.
• Diabetic patients receiving empagliflozin after acute myocardial infarction presented with improved endothelial glycocalyx thickness and percentage global longitudinal strain, increased flow-mediated dilation, preserved pulse wave velocity, and reduced intercellular adhesion molecule 1 circulating levels at 4- and 12-month follow-ups.

## Visual Abstract

**Figure:**
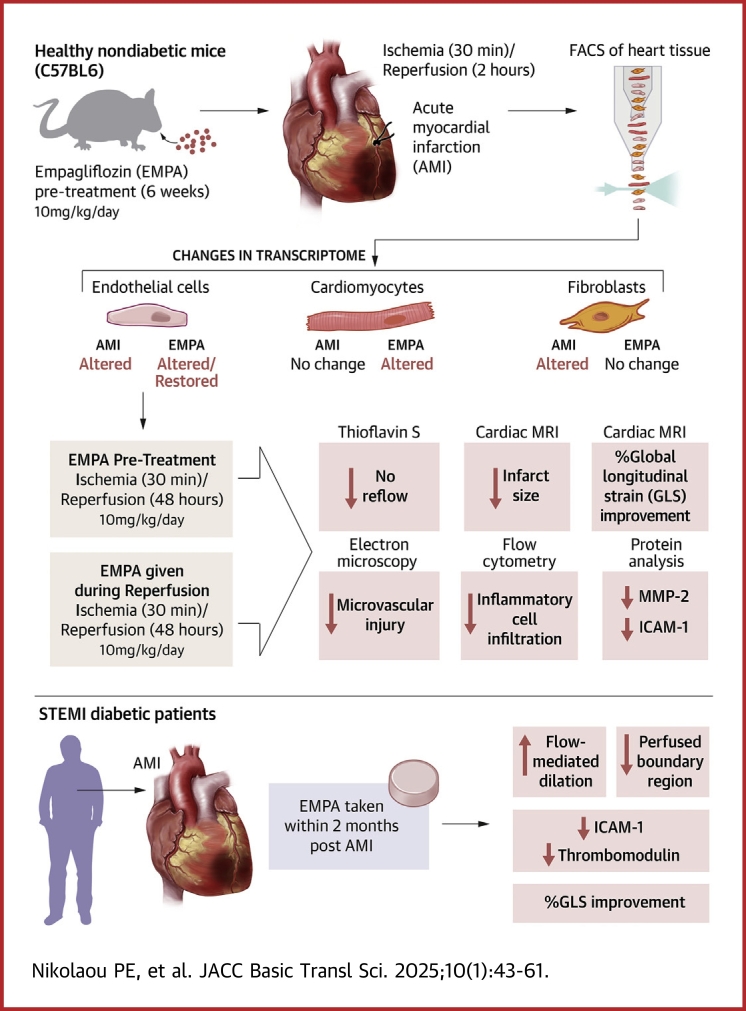

Significant advances in the past decades have led to a notable reduction in morbidity and mortality following ST-segment elevation myocardial infarction (STEMI).^1^ Despite revascularization that ultimately reduces infarct size,^2^ injurious processes for cardiomyocytes and the coronary vasculature are activated during reperfusion.^3^^,^^4^ Upon blood flow restoration, the coronary microvascular integrity is affected, leading to edema, intramyocardial hemorrhage, and increased infarct size.^5^ No-reflow is severe myocardial malperfusion despite restoration of coronary patency.^6^ It is a manifestation of severe microvascular injury, occurs in up to 50% of patients with STEMI, and is associated with left ventricular (LV) function impairment.^6^, ^7^, ^8^ Every 1% increase in microvascular obstruction leads to a 14% increase in all-cause death within 1 year and 8% increase in 1-year heart failure hospitalization.^8^

Clinical trials indicate that specific populations may benefit from sodium-glucose cotransporter-2 inhibitors (SGLT-2Is). Empagliflozin (EMPA) treatment after acute myocardial infarction (AMI) reduced the risk for first and total heart failure hospitalizations^9^ and the risk of heart failure in patients with LV dysfunction or congestion.^10^ However, it did not reduce the composite endpoint of hospitalization for heart failure or death from any cause.^10^^,^^11^ Dapagliflozin administration after AMI altered cardiometabolic outcomes with no significant impact on the primary endpoint.^12^ However, no preclinical or clinical evidence is available regarding the potential benefits of SGLT-2Is on microvascular injury in a translational setting after AMI.

Therefore, we aimed to explore the effect of EMPA treatment administered either before or after AMI against coronary microvascular injury. To this end, we investigated: 1) EMPA’s effect on the transcriptome of cardiac cell types in vivo, including endothelial cells (ECs), which contribute to microvascular injury;^5^^,^^8^ 2) EMPA’s effect on vessel integrity based on no-reflow, with histology, electron microscopy, and flow cytometry–based evaluation of inflammatory cell infiltration in the ischemic heart; 3) infarct size and cardiac function by implementing state-of-the-art cardiac magnetic resonance imaging; 4) the establishment of a translational protocol for EMPA’s administration early after reperfusion, in which the coronary vascular and cardiac protection pertains; and 5) proof-of concept clinical evidence for endothelial and cardiovascular parameters in patients with type 2 diabetes and high cardiovascular risk who received EMPA after AMI to facilitate future investigations on a larger scale.

## Methods

Detailed methodology is described in the Supplemental Methods.

### Mice

All animal procedures conformed to the Presidential Decree 56/2013 for the protection of the animals used for scientific purposes, in harmonization with the European Directive 2010/63/EU. The experimental protocols were described and approved by the competent Veterinary Service of the Prefecture of Athens (license protocol nos. 557314/30-07-2020 and 179489/13-02-2023). The animal procedures, performed in Radboud University Medical Center (UMC) under project license AVD10300 2023 16675, were authorized by the Central Authority for Scientific Procedures on Animals in the Netherlands. A total of 155 C57BL/6 male mice, 8-12 weeks old, were used for experimentation, 95 in the Biomedical Research Foundation of the Academy of Athens and 60 in the Preclinical Imaging Center of Radboud UMC. Surgical procedures and interventions were performed in compliance with the guidelines,^13^ that is, mice randomization was performed in all experiments, surgical procedures and analysis of the experimental data were performed in a blinded manner which was ensured with the use of Castor EDC (v2022.4.1.3), and sample size was defined a priori (Supplemental Figures 1 and 2). Exclusion criteria were defined a priori as the absence of ST-segment elevation in electrocardiography and the rupture of the suture/vessel during the surgical procedure for AMI induction.

### In vivo experimental protocols

#### First series of experiments

EMPA’s effect on the transcriptome of different cell populations was investigated. Daily oral administration of EMPA (10 mg/kg/d) or vehicle (5% dimethylsulfoxide in sterile normal saline solution) was performed for 6 weeks based on our previous study regarding the cardioprotective effect of EMPA in nondiabetic mice,^14^ which corresponds to 25 mg/d in humans.^14^, ^15^, ^16^ Ischemia was induced by left anterior descending coronary artery ligation. Mice (n = 30, n = 10 per group, were randomized into:

1. Sham: Daily oral administration of vehicle and open-chest operation.
2. Control-AMI: Daily oral administration of vehicle, 30 minutes of ischemia, and 2 hours of reperfusion.
3. EMPA–Pre-AMI: Daily oral administration of EMPA and 30 min of ischemia and 2 h of reperfusion.

To isolate sufficient quantity of high-quality RNA, we pooled together 2 hearts per sample and performed 4 to 5 independent experiments per cell population per group.^17^ Cardiomyocytes were isolated,^18^ and ECs and fibroblasts were sorted for RNA sequencing as described in the Supplemental Methods (Supplemental Figure 3).

#### Second series of experiments

Mice (n = 18) were randomized as in the first series of experiments. After 2 hours of reperfusion, the ischemic LV was excised to validate the results of the RNA sequencing with real time polymerase chain reaction and protein analysis for selected genes.

#### Third series of experiments

Mice (n = 9) received 10 mg/kg EMPA or vehicle (n = 3) orally, and heparinized plasma was collected for the determination of EMPA circulating levels 1 hour after oral administration. This experiment was repeated twice within 48 hours.

#### Fourth series of experiments

We investigated no-reflow with the use of Thioflavin S staining,^19^^,^^20^ and determined infarct size with the use of 2,3,5-triphenyl tetrazolium chloride staining^16^^,^^21^ and cardiac magnetic resonance imaging.^20^ We also examined cardiac function by means of cardiac magnetic resonance at 48 hours of reperfusion.^20^ Mice (n = 60) were randomized into 4 groups as follows:

1. Sham (n = 8): Daily oral administration of vehicle before and after open-chest operation.
2. Control-AMI (n = 18): Daily oral administration for 6 weeks of vehicle before and after 30 minutes of ischemia and 48 hours of reperfusion.
3. EMPA–Pre-AMI (n = 18): Daily oral administration of EMPA (for 6 weeks) and vehicle (once daily) for 48 hours after 30 minutes of ischemia.
4. EMPA–Post-AMI (n = 16): Daily oral administration of vehicle (for 6 weeks). EMPA (10 mg/kg/d) was given orally 60 minutes (1 hour) after reperfusion and was continued once daily for 48 hours.

#### Fifth series of experiments

We repeated the protocol in the fourth series (n = 6-9 per group). At 48 hours after reperfusion, the heart was collected for flow cytometry^22^^,^^23^ to examine inflammatory cell infiltration, and part of the ischemic myocardium was snap-frozen for protein analysis. The gating strategy is depicted in Supplemental Figure 4.

### Human study

Patients with type 2 diabetes and high cardiovascular risk were recruited from the cardiometabolic outpatient clinic of Attikon Hospital.^24^ The investigation conformed to the principles outlined in the Declaration of Helsinki. The study protocol was approved by the University General Hospital “Attikon” Institutional Review Board before recruitment initiation. In addition, all participants gave their written informed consent.

Patients of this study were included in an ongoing randomized prospective study investigating the effects of glucagon-like peptide 1 receptor agonists (liraglutide), SGLT-1Is (EMPA), and their combination on endothelial glycocalyx, arterial function, and myocardial work index in patients with type 2 diabetes mellitus after 12 months of treatment (The Cardiovascular Effect of GLP-1 Agonist, SGLT2 Inhibitor and Their Combination; NCT03878706). As a second-line treatment after metformin, patients received basal insulin or 25 mg oral EMPA once daily for 12 months. Patient enrollment was performed from November 2017 to September 2022, and subgroup analysis was based on the criterion that the initiation of the EMPA treatment was within 2 months after STEMI. Out of the pool of 120 patients, a total of 24 patients for the EMPA group and 18 for the insulin group were matched for age, sex, and glycosylated hemoglobin (HbA_1c_) and underwent clinical, vascular, and 2-dimensional echocardiography examinations at baseline and 4- and 12-month follow-ups, at which time points blood sampling was also performed. Perfused boundary region, flow-mediated dilation of the brachial artery, the carotid-femoral pulse wave velocity, and circulating endothelial markers were determined. All studies were analyzed by 2 observers blinded to clinical and laboratory data.

### Statistical analysis

Bioinformatic analyses for RNA sequencing data are described in detail in the Supplemental Methods. Bar plots are presented as mean ± SD. Normality assessment was performed with Anderson-Darling, d’Agostino, Pearson, Shapiro-Wilk, Kolmogorov-Smirnov tests. One-way analysis of variance (ANOVA) with Tukey post hoc test for multiple pairwise comparisons was applied for in vivo studies. Kruskal-Wallis with Dunn multiple comparisons test was applied for the histology scores assessment and percentage infarct/LV measurement with 2,3,5-triphenyl tetrazolium chloride staining because the EMPA–Post-AMI data were not normally distributed. Two-way repeated-measures ANOVA and Tukey post hoc comparisons were performed on data from patients receiving EMPA or insulin. No assumption of equal variability of differences was performed, and data were corrected with Greenhouse-Geisser correction. Correlation analysis was performed with the use of parametric (Pearson) or nonparametric (Spearman) correlation coefficients, based on data distribution, including linear regression to estimate the slope with SE. To identify whether the regression line differs from the line of identity for the infarct size determined by cardiac magnetic resonance imaging and staining, we used the concordance correlation coefficient introduced by Lin in 1989^25^ to assess the agreement between the 2 variables (slopes).^26^ A value of *P* ≤ 0.05 was considered to be statistically significant. Statistical analyses and graph preparation were performed using GraphPad Prism 8.5 analysis software (GraphPad Software). The presence of outlying values was identified with the use of GraphPad Prism analysis software using the ROUT method and Q = 1%.

## Results

### EMPA pretreatment reverses AMI-induced alterations in the transcriptome of ECs

**Figure 1:**
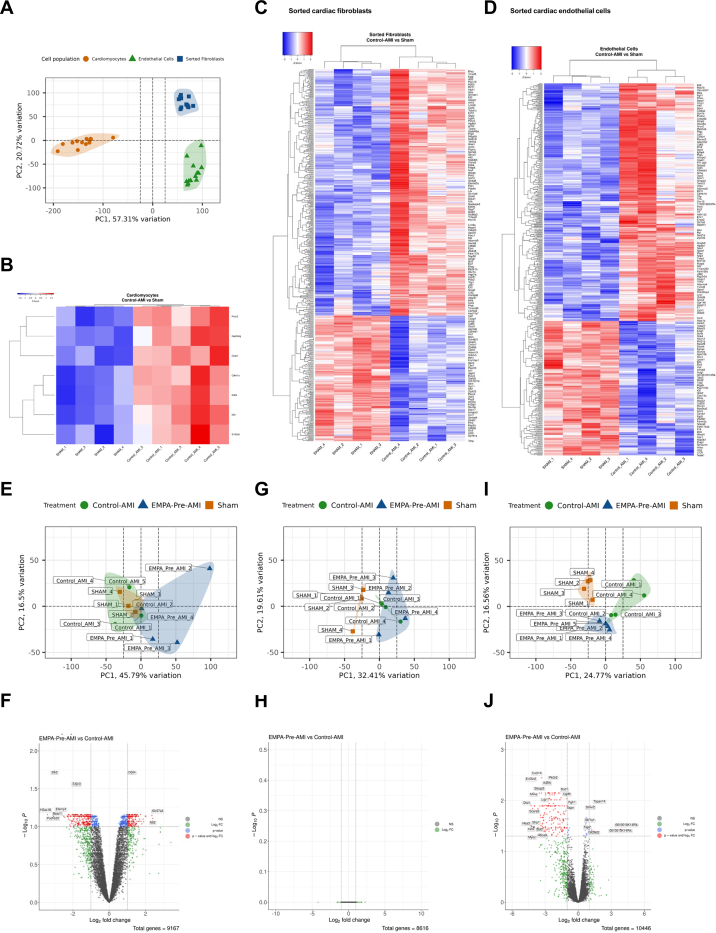
Effect of AMI and EMPA on Cardiomyocytes, Fibroblasts, and EC Transcriptome 2 Hours After Reperfusion (A) Principal component (PC) analysis plot with the relative gene expression for all samples indicating the discrimination of the cell populations. Heatmap gene expression analyses depicting the significant differences between the Sham and the Control-AMI groups in (B) isolated cardiomyocytes, (C) sorted fibroblasts, and (D) sorted ECs. PC analyses showing the discrimination among the Sham, Control-AMI, and EMPA–Pre-AMI groups in (E) cardiomyocytes, (G) sorted fibroblasts, and (I) sorted ECs. Volcano plots indicating the significant genes as blue dots and the significant and deregulated genes as red dots between the Control-AMI and EMPA–Pre-AMI groups in (F) cardiomyocytes (FDR: <0.10), (H) sorted fibroblasts (FDR: <0.10), and (J) sorted ECs (FDR: <0.05). AMI = acute myocardial infarction; EC = endothelial cells; EMPA = empagliflozin; FDR = false discovery rate.

The distinct gene expression profile of ECs, fibroblasts, and cardiomyocytes was indicated by the principal component analysis and mean squared displacement plots (Figure 1A, Supplemental Figure 5). In cardiomyocytes, 7 significant genes (false discovery rate [FDR]: <0.01) between the Sham and Control-AMI groups were identified (Figure 1B). Those genes did not correspond to significant pathways. The fibroblast transcriptome was affected by AMI with 571 significant genes (FDR: <0.05) between Sham and Control-AMI (Figure 1C). Those genes corresponded to 5 pathways (FDR: <0.10) including “Interleukin-6 signaling” and “Gap junction degradation” (Supplemental Figure 6A). The EC transcriptome was significantly deregulated by AMI with 576 significant genes (FDR: <0.05) between Sham and Control-AMI (Figure 1D), corresponding to 15 pathways (FDR: <0.05). The deregulated pathways involved extracellular matrix (ECM) organization, cell interaction via junctions, and the proteolytic degradation of basement membranes (Supplemental Figure 6B).

Cardiomyocyte transcriptome indicated 3 significant genes (FDR: <0.05) and 811 genes (FDR: <0.1) between Control-AMI and EMPA–Pre-AMI (Figures 1E and 1F), which were mapped to pathways related to mitochondrial metabolism (FDR: <0.10) (Supplemental Figure 7). We assumed that this is an EMPA-specific effect, which we have previously addressed.^14^ EMPA did not significantly alter the transcriptome of fibroblasts (Figures 1G and 1H).

**Figure 2:**
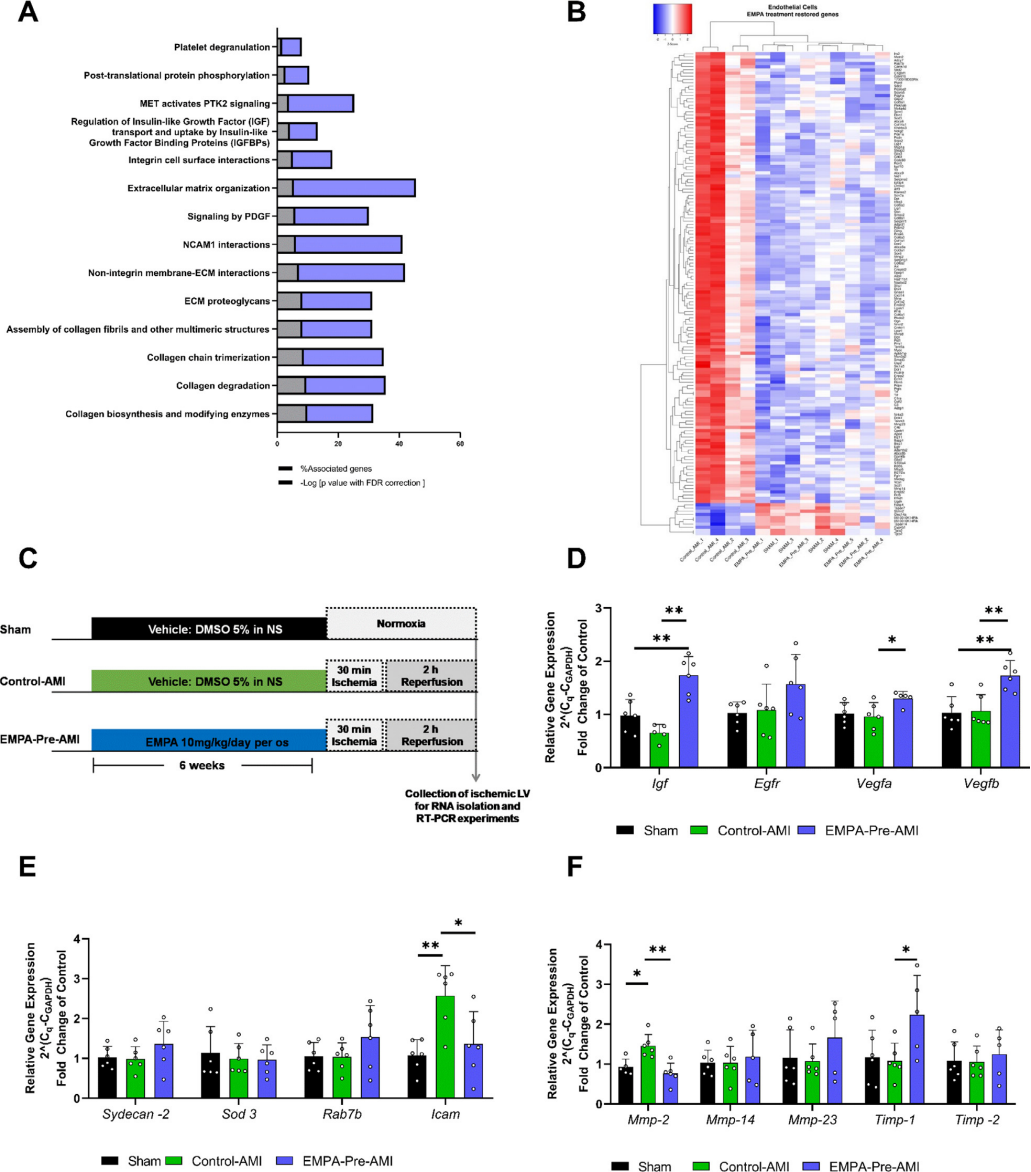
EMPA Pretreatment Reverses the Deregulated Transcriptome by AMI in ECs (A) Bar plots indicating the significant Reactome pathways between the Control-AMI and EMPA–Pre-AMI group 2 hours after reperfusion in sorted ECs with FDR <0.05. (B) Heatmap analysis demonstrating the 150 genes that are significantly deregulated by AMI and restored by EMPA pretreatment. (C) Experimental protocol for RNA sequencing data validation. Relative gene expression 2 hours after reperfusion as 2^−(Cq-C_Gapdh_^^)^ (fold change of Control-AMI) for (D) growth factor genes and regulators, (E) genes related to cell adhesion, and (F) genes related to extracellular matrix and collagen degradation. Data are presented as mean ± SD (n = 5-6 per group; 1-way analysis of variance with Tukey post hoc test: ∗*P* < 0.05; ∗*P* < 0.05; ∗∗*P* < 0.01. DMSO = dimethylsulfoxide; FDR = false discovery rate; other abbreviations as in Figure 1.

An effective discrimination between Control-AMI and EMPA–Pre-AMI was observed only in the EC population (Figure 1I) with 211 significant genes (FDR: <0.05) (Figure 1J). EMPA treatment affected 13 of the 15 AMI-deregulated pathways, indicating that it restored the EC transcriptome in vivo (Figure 2A, Supplemental Figure 6B). Moreover, 150 genes were significantly deregulated by AMI and restored by EMPA treatment (Figure 2B), and out of this pool we selected growth factor genes as regulators of EC survival and genes related to cell adhesion or collagen degradation/ECM to validate in an additional cohort of mice (Figure 2C). Vascular endothelial growth factor B (*Vegfβ*) and insulin-like growth factor-1 (*Ιgf-1*) expression were increased by EMPA pretreatment (Figure 2D). EMPA attenuated the AMI-induced increase of intercellular adhesion molecule-1 (*Icam-1*) and metalloproteinase-2 (*Mmp-2*) genes after 2 hours of reperfusion (Figure 2E), and it increased the expression of the tissue inhibitor of the metalloproteinases-1 (*Timp-1*) gene (Figure 2F), validating the RNA sequencing data.

### EMPA treatment before and after AMI reduces no-reflow and improves vascular integrity

**Figure 3:**
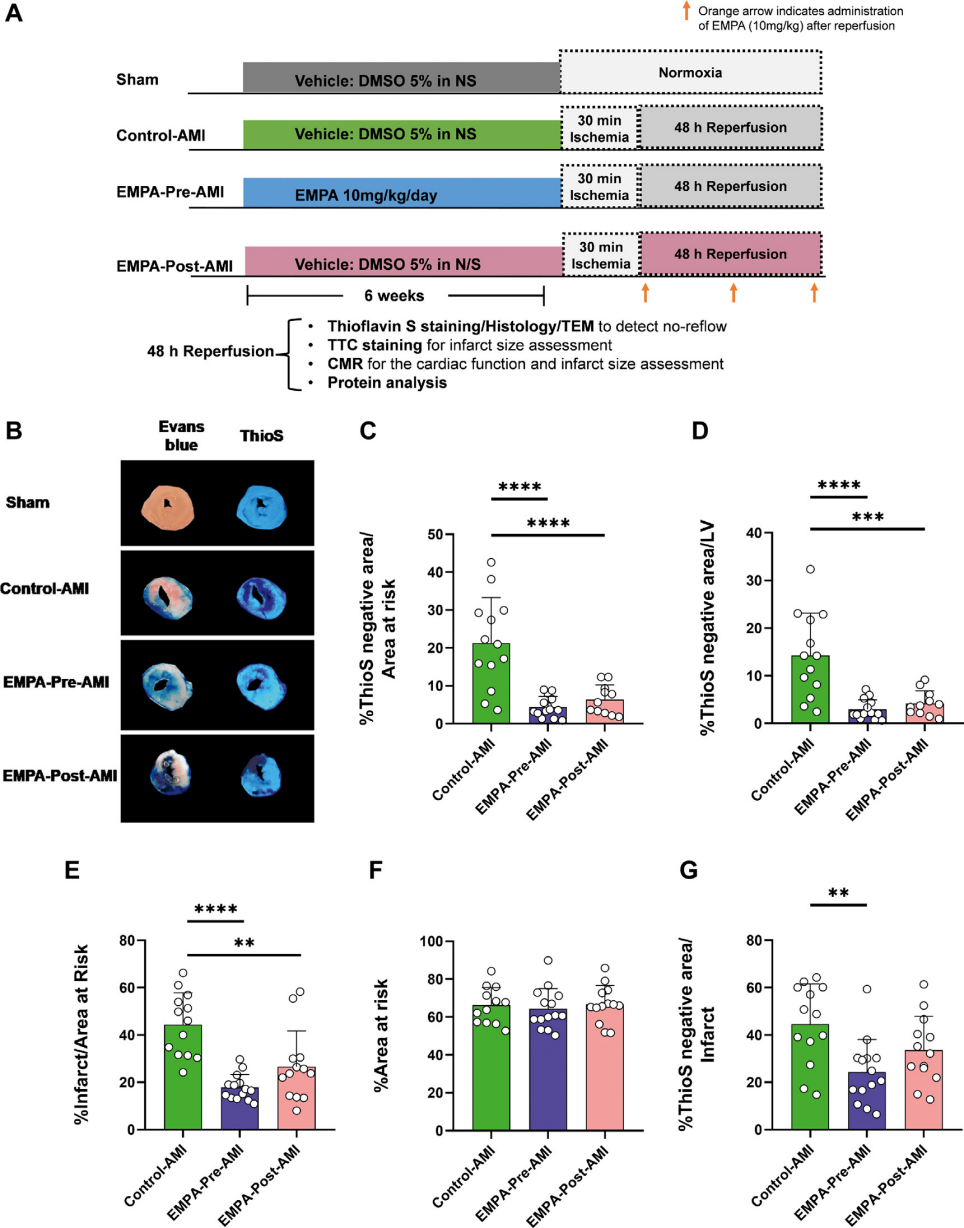
EMPA Treatment Before and After AMI Induction Reduces No-Reflow and Infarct Size (A) Schematic representation of the experimental protocol. (B) Representative images of Evans Blue and Thioflavin S staining 48 hours after reperfusion. Bar plots for (C) percentage Thioflavin S (%ThioS)–negative area to area at risk, (D) %ThioS–negative area to left ventricle (LV), (E) percentage infarct to area at risk, (F) percentage area at risk to LV, and (G) %ThioS–negative area to infarct size. Data are presented as mean ± SD (n = 8-14 per group; 1-way analysis of variance with Tukey post hoc test: ∗∗*P* < 0.01; ∗∗∗*P* < 0.001; ∗∗∗∗*P* < 0.0001. DMSO = dimethylsulfoxide; NS = normal saline solution; other abbreviations as in Figure 1.

The transcriptomic data illustrate effects of EMPA on ECs that are consistent with coronary endothelial protection, but further evidence is needed to support these findings. Therefore, we subsequently hypothesized that EMPA treatment either before or after AMI may alleviate microvascular injury. The experimental protocol was designed based on the time point when microvascular injury is detected in rodents (48 hours after reperfusion)^20^ and the circulating levels of EMPA (Figure 3A). EMPA levels reached approximately 400 ng/mL 1 hour after the oral administration (Supplemental Table 5), which is similar to C_max_ levels observed in humans treated with 25 mg/d EMPA.^27^ Therefore, EMPA was given 1 hour after reperfusion to reach this concentration at 2 hours after reperfusion, when the EC transcriptome was affected.

No-reflow to the area at risk and no-reflow to the LV were significantly improved in both EMPA–Pre-AMI and EMPA–Post-AMI groups (Figures 3B to 3D), indicating reduced microvascular injury 48 hours after AMI (Supplemental Table 6). In line with no-reflow reduction, infarct size to area at risk was significantly reduced in EMPA–Pre-AMI and EMPA–Post-AMI (Figure 3E). Area at risk did not differ among the groups, indicating that a similar ischemic insult was induced (Figure 3F). No-reflow to the infarct size was significantly reduced in EMPA–Pre-AMI but not in EMPA–Post-AMI (Figure 3G).

**Figure 4:**
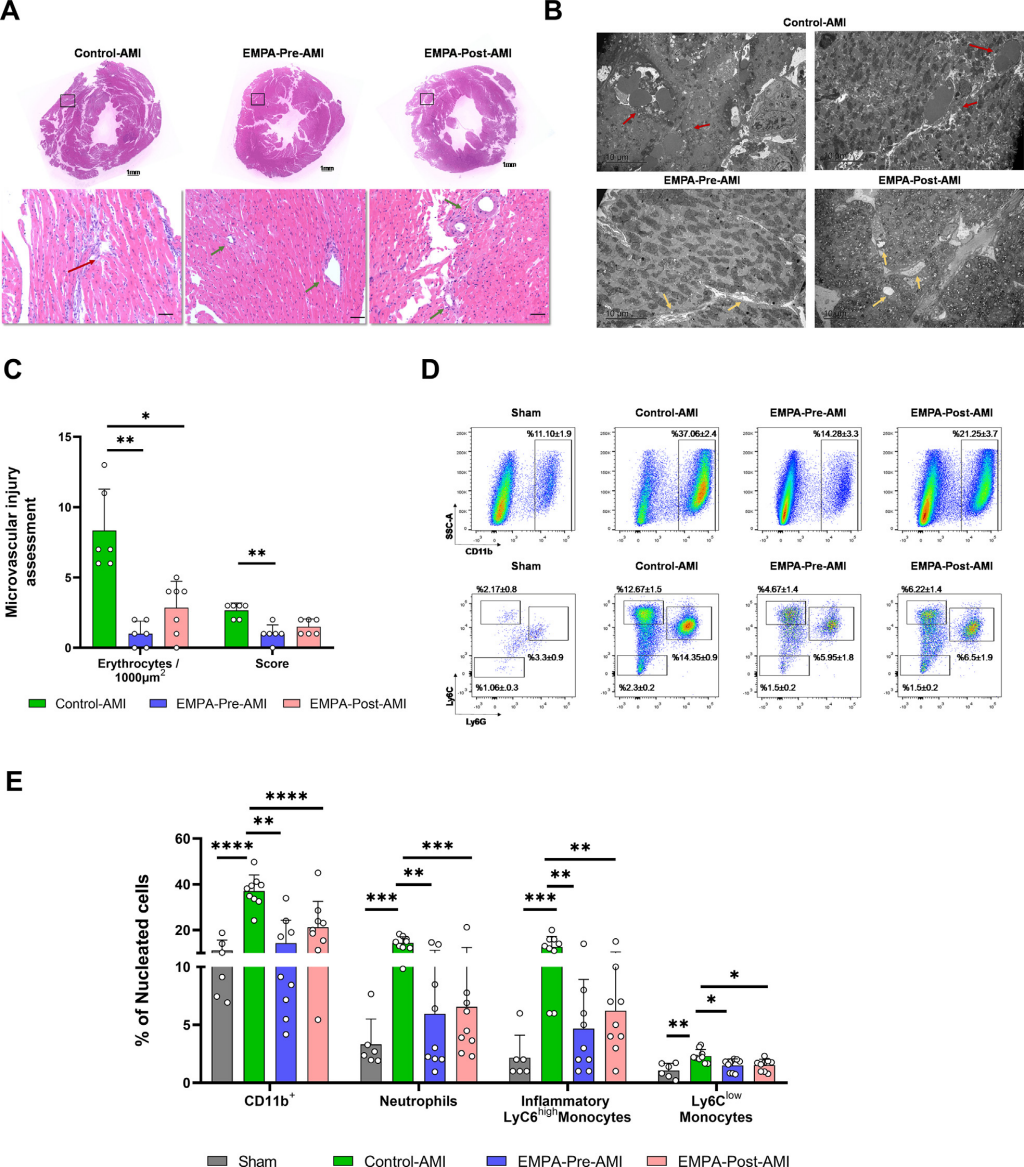
EMPA Treatment Before and After AMI Improves Capillary Integrity and Prevents Infiltration of Inflammatory Cells in the Infarcted Heart (A) Representative images of hematoxylin-eosin staining. Squares indicate the area of focus. Red arrow denotes destroyed/disrupted vessel, and green arrows denote vessels with structural integrity. (B) Representative electron micrographs indicating intramyocardial hemorrhage. Red arrows depict extravasation of erythrocytes, indicating intramyocardial hemorrhage and capillaries clogged by erythrocytes. Yellow arrows denote intact capillaries. (C) Quantification of erythrocytes in transition electron microscopy images and microvascular injury score in hematoxylin-eosin images for the respective groups. (D) Representative images of flow cytometric analysis of the inflammatory cells in the infarcted heart. (E) Bar plots with the percentage of each cell population expressed as a percentage of the nucleated cells. Data are presented as mean ± SD (n = 6-9 per group; 1-way analysis of variance with Tukey post hoc test: ∗*P* < 0.05; ∗∗*P* < 0.01; ∗∗∗*P* < 0.001; ∗∗∗∗*P* < 0.0001. Abbreviations as in Figure 1.

The effect of EMPA before and after AMI on microvascular injury was further assessed based on histologic ultrastructure preservation of the capillaries and transition electron microscopy micrographs in the no-reflow area (Figures 4A and 4B, Supplemental Figure 8). Extravasation of erythrocytes, indicating intramyocardial hemorrhage, and intact capillaries clogged by erythrocytes were more prominent in the Control-AMI group. In the EMPA–Pre-AMI and EMPA–Post-AMI groups, less microvascular injury was observed, as indicated by the reduced number of erythrocytes and the preserved percentage of intact capillaries, suggesting better endothelial cell junction integrity (Figure 4C).

### EMPA treatment before and after AMI significantly attenuates inflammatory cell infiltration in the infarcted heart

Leukocyte recruitment and adhesion to the endothelium have been described as contributors to no-reflow by participating in the formation of microthrombi that further exacerbate inflammatory cell infiltration.^28^^,^^29^ Our findings suggest that EMPA before and after AMI suppresses the inflammatory cell infiltration in the infarcted heart. Control-AMI induced the overrepresentation of CD11b^+^ cells, neutrophils, inflammatory LyC6^high^ monocytes, and LyC6^low^ monocytes in the infarcted heart 48 hours after reperfusion. EMPA treatment before and after AMI significantly reduced the infiltration of CD11b^+^ cells, neutrophils, and inflammatory LyC6^high^ monocytes (Figures 4D and 4E). No difference was observed in T and B cells or the CD4/CD8 T-cell ratio in the heart (Supplemental Figures 9A to 9C).

### EMPA reduces infarct size and restores global cardiac function

**Figure 5:**
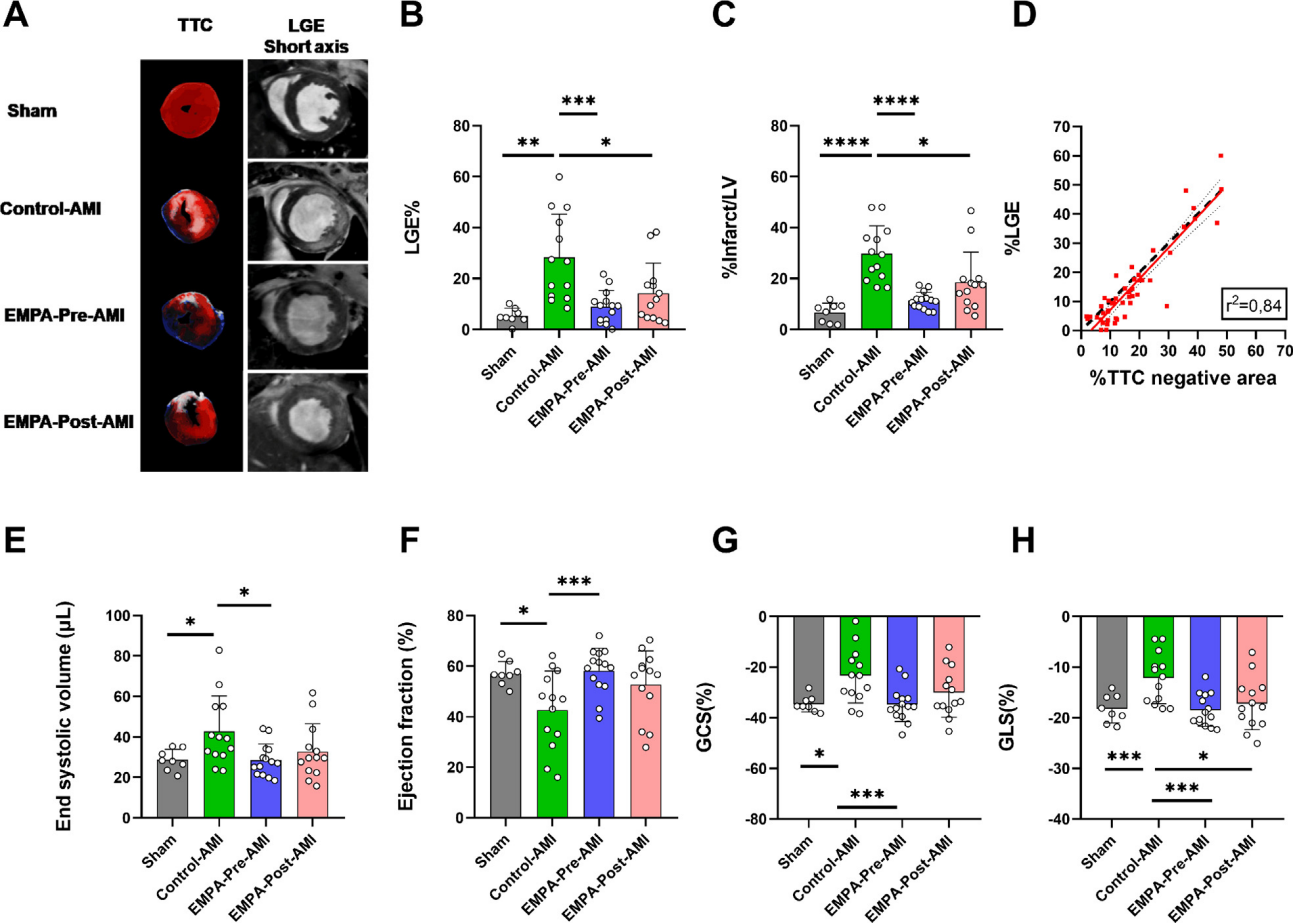
EMPA Treatment Before and After AMI Reduces Infarct Size and Improves Cardiac Function (A) Representative images of 2,3,5-triphenyl tetrazolium chloride (TTC) staining and late gadolinium enhancement (LGE) in short axis for each experimental group 48 hours after reperfusion. (B) Percentage LGE infarction determined by cardiac magnetic resonance imaging and (C) infarct/left ventricle (LV) via staining. (D) Linear regression (solid red line) and the line of identity (dashed line) comparing the infarct size values determined via staining and %LGE. Bar plots indicating (E) end-systolic volume, (F) LV ejection fraction, (G) global circumferential strain (GCS), and (H) global LV longitudinal strain (GLS). Data are presented as mean ± SD (n = 8-14 per group; 1-way analysis of variance with Tukey post hoc test: ∗*P* < 0.05; ∗∗*P* < 0.01; ∗∗∗*P* < 0.001; ∗∗∗∗*P* < 0.0001. Abbreviations as in Figure 1.

Cardiac magnetic resonance imaging evaluation confirmed the cardioprotective effect of EMPA treatment before and after AMI in terms of infarct size and cardiac function. Late gadolinium enhancement analysis in cardiac magnetic resonance imaging indicated that EMPA before and after AMI significantly reduced infarct/LV compared with Control-AMI (Figures 5A and 5B). Similar results were obtained in 2,3,5-triphenyl tetrazolium chloride staining (Figure 5C). Infarct size determined by cardiac magnetic resonance imaging and staining indicated a linear association with a slope ± SE of 1.062 ± 0.068 and Pearson *r* of 0.92. The concordance correlation coefficient (0.89) indicated that the regression line (solid red line) and the line of identity (dashed black line) do not differ significantly (Figure 5D). EMPA before AMI completely restored global cardiac function, as shown by the preserved LV ejection fraction**,** end-systolic volume, cardiac output, stroke volume, and mass (Figures 5E and 5F, Supplemental Table 8). Both groups improved global circumferential strain (Figure 5G) and completely restored % global left ventricular longitudinal strain (%GLS) (Figure 5H).

### EMPA reduces ICAM-1 and MMP-2 expression and STAT-3 phosphorylation at late reperfusion

**Figure 6:**
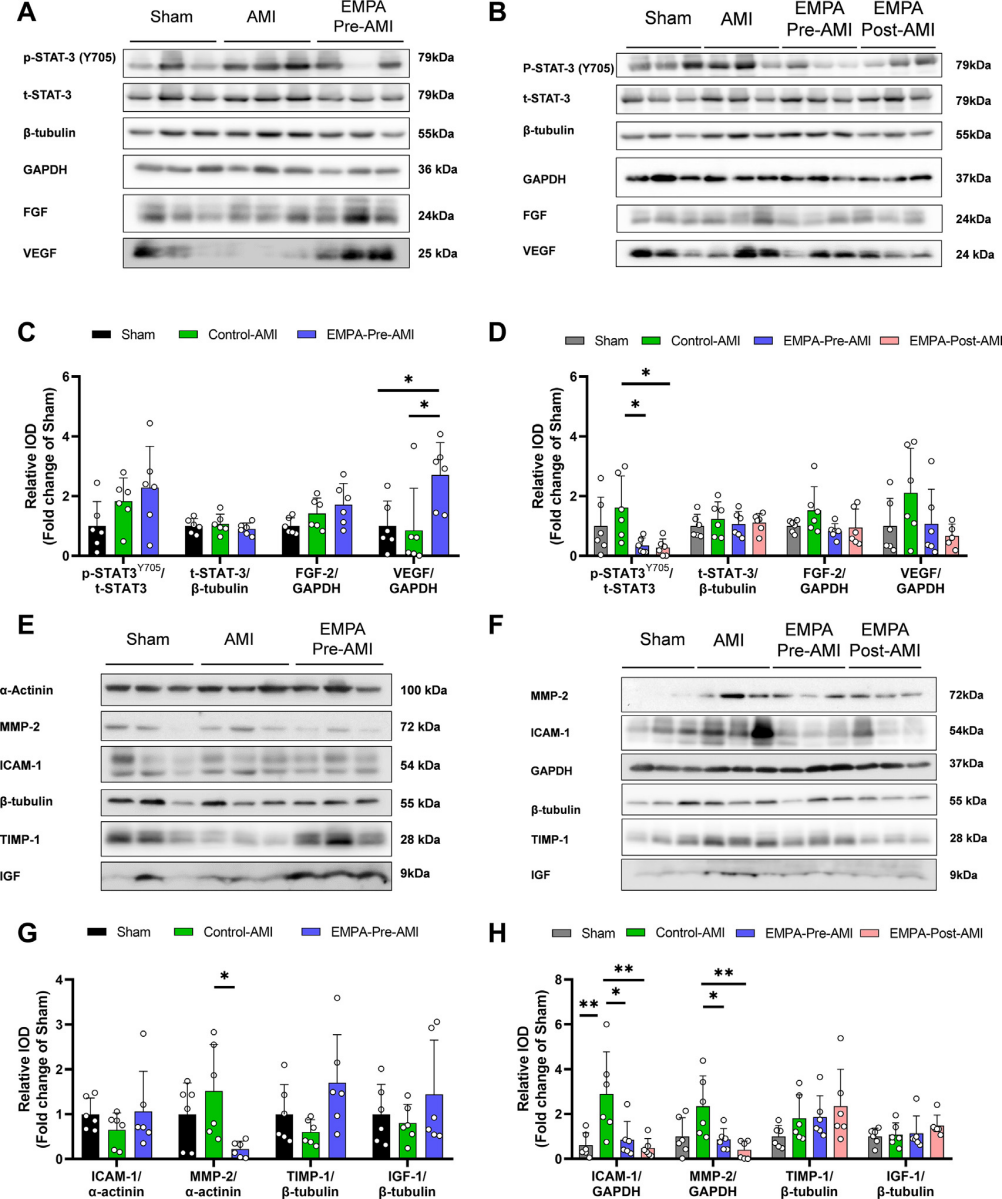
EMPA Reduces ICAM-1 and MMP-2 Expression and STAT-3 Phosphorylation at Late Reperfusion Representative Western blots (A) 2 hours and (B) 48 hours after reperfusion. Relative densitometric graphs after normalization to Sham of (C) p-(Y703)STAT-3/t-STAT-3, t-STAT-3/β-tubulin, FGF-2/GAPDH, and VEGF/GAPDH 2 hours after reperfusion and (D) p-(Y703)STAT-3/t-STAT-3, t-STAT-3/β-tubulin, FGF-2/GAPDH, and VEGF/GAPDH 48 hours after reperfusion. Representative Western blots (E) 2 hours and (F) 48 hours after reperfusion. Relative densitometric graphs after normalization to Sham of (G) ICAM-1/α-actinin, MMP-2/α-actinin, TIMP-1/β-tubulin, and IGF-1/β-tubulin at 2 hours of reperfusion and (H) ICAM-1/GAPDH, MMP-2/GAPDH, TIMP-1/β-tubulin, and IGF-1/β-tubulin at 48 hours of reperfusion. Dots represent biological replicates. Results are presented as mean ± SD (n = 6 per group; 1-way analysis of variance with Tukey post hoc test: ∗*P* < 0.05; ∗∗*P* < 0.01. AMI = acute myocardial infarction; EMPA = empagliflozin; FGF = fibroblast growth factor 2; ICAM = intercellular adhesion molecule; IGF = insulin-like growth factor; MMP = matrix metalloproteinase; STAT = signal transducer and activator of transcription; TIMP = tissue inhibitor of the metalloproteinase; VEGF = vascular endothelial growth factor.

To gain further insights into the mechanism of EMPA-mediated cardioprotection in terms of microvascular injury and infarct size reduction, we further performed protein analysis 2 hours and 48 hours after reperfusion. We have previously shown that EMPA pretreatment increases the phosphorylation and activation of signal transducer and activator of transcription 3 (pSTAT-3; Y705) at the 10th minute of reperfusion in diabetic and healthy nondiabetic mice,^14^^,^^15^ although this increase does not reach statistical significance 2 hours after reperfusion using the same experimental protocol applied herein.^2^ In parallel, increased protein levels of growth factor signaling, including fibroblast growth factor 2 (FGF-2) and VEGF have been detected by EMPA pretreatment.^14^^,^^16^ In this study, AMI and EMPA pretreatment did not affect STAT-3 phosphorylation and FGF-2 levels 2 hours after reperfusion (Figures 6E and 6G), confirming our previous data, that this pathway is activated by EMPA at early (10 min) rather than late reperfusion (2 h).^14^ We found that VEGF was increased 2 hours after reperfusion in EMPA–Pre-AMI compared with Sham and Control-AMI (Figure 6E and 6G), indicative of preserved EC survival, as we have previously shown.^14^ Molecular signaling 48 hours after reperfusion showed that both EMPA–Pre-AMI and EMPA–Post-AMI significantly reduced STAT-3 phosphorylation compared with Control-AMI without altering growth factor levels and STAT-3 expression (Figure 6F and 6H). This finding suggests that EMPA may confer different signaling during the time course evolution of AMI.

Subsequently, we focused on pathways related to the ECs and microvascular injury, and we evaluated the expression levels of proteins related to our transcriptomic findings. At 2 hours after reperfusion, EMPA pretreatment led to a significant reduction of MMP-2 and a marginally significant increase (*P* = 0.05) in TIMP-1 protein levels, whereas no alterations were detected in ICAM-1 (Figures 6E and 6G). At 48 h, AMI led to a significant increase in ICAM-1 and to a marginally significant increase in MMP-2 (*P* = 0.05), which were significantly attenuated in EMPA–Pre-AMI and EMPA–Post-AMI (Figures 6F and 6H). Insulin-like growth factor I levels were not altered by AMI or EMPA treatment at either time point. These findings confirmed the transcriptomic alterations in ECs in the murine myocardium, indicating that EMPA’s cardioprotection both before and after AMI could be linked to improved ECM remodeling and reduced immune cell adhesion.

### EMPA treatment after STEMI in patients with diabetes improves endothelial and cardiac dysfunction altering the circulating markers thrombomodulin and ICAM-1

**Figure 7:**
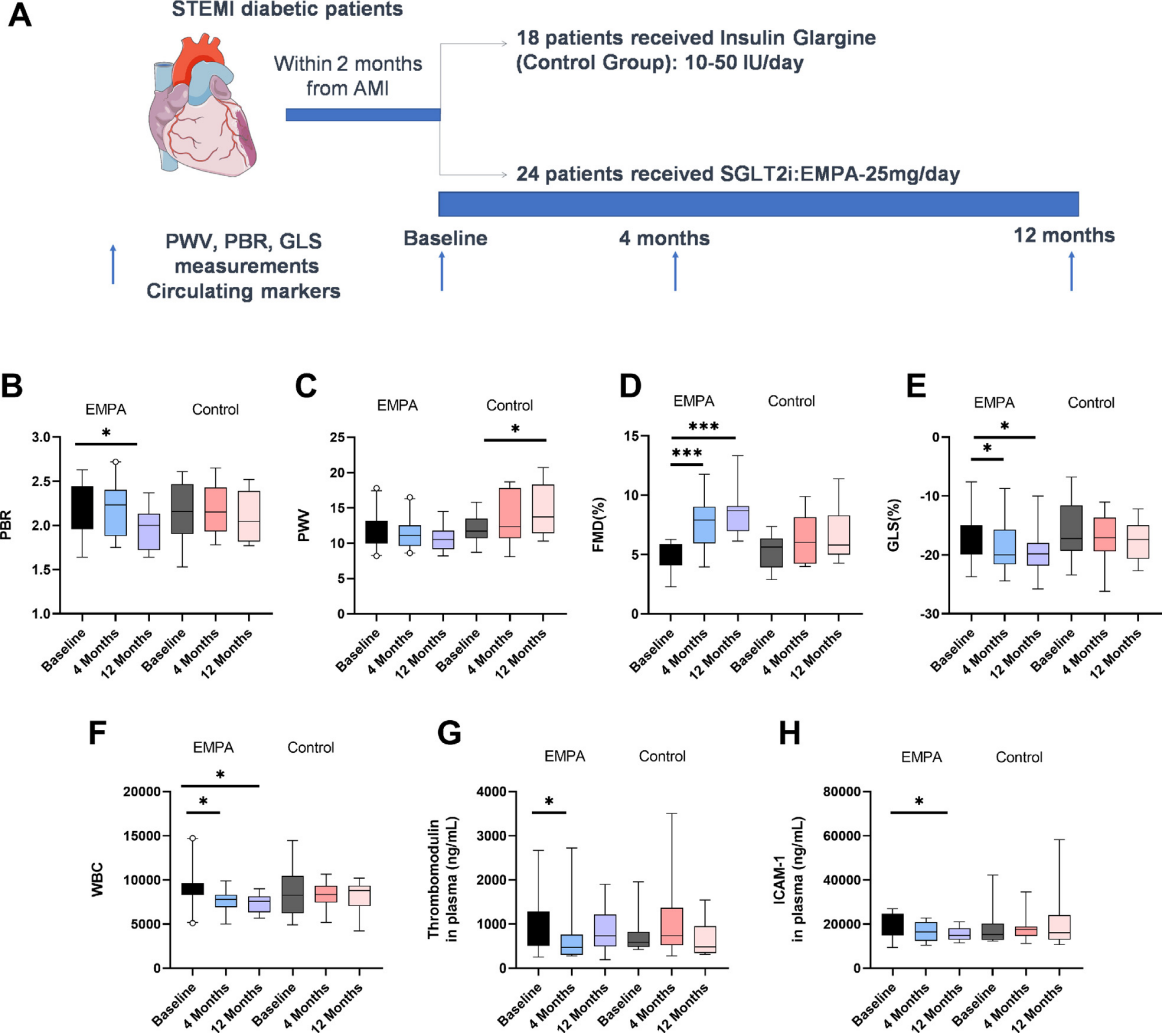
EMPA Treatment Within 2 Months of STEMI Improves Endothelial and Cardiac Dysfunction Altering the Circulating Markers Thrombomodulin and ICAM-1 (A) Schematic representation of the clinical protocol. EMPA was given within 2 months after STEMI, and clinical parameters were evaluated at 4 months and 12 months after inclusion. Box whisker plots with 5th to 95th percentiles indicating (B) perfused boundary region (PBR), (C) pulse-wave velocity (PWV), (D) percentage flow-mediated dilation (FMD), (E) percentage global longitudinal strain (GLS), (F) white blood cell count (WBC), (G) thrombomodulin levels in plasma (ng/mL), and (H) ICAM-1 levels in plasma (mg/mL) at 4- and 12-month follow-ups. Data are presented as mean ± SD (n = 24 for EMPA and n = 18 for Control; 2-way repeated-measures analysis of variance with Tukey post hoc test: ∗*P* < 0.05; ∗∗∗*P* < 0.001. % GLS = % global longitudinal strain; EMPA = empagliflozin; FMD% = flow-mediated dilation of the brachial artery; ICAM = intercellular adhesion molecule.

Baseline characteristics for patients are depicted in Supplemental Table 10, and no significant differences were observed between groups. All patients achieved an HbA_1c_ <7% after treatment. EMPA treatment after STEMI (Figure 7A) significantly reduced perfused boundary region at 12-month follow-up and prevented the aggravation of pulse wave velocity at both 4- and 12-month follow-ups (Figures 7B and 7C). EMPA treatment significantly improved flow-mediated dilation of the brachial artery and %GLS at 4- and 12-month follow-ups (Figures 7D and 7E). EMPA-treated patients presented with significantly reduced white blood cell count (WBC) at both time points (Figure 7F), as well as reduced thrombomodulin and ICAM-1 levels in plasma (Figures 7G and 6H).

In the EMPA group, the percentage change of perfused boundary region at 4 and 12 months was significantly correlated with the percentage change of %GLS (*r* = −0.550; *P* = 0.030; and *r* = −0.650; *P* = 0.020; respectively). The percentage change of thrombomodulin at 4 months correlated with the corresponding changes of pulse-wave velocity (*r* = 0.571; *P* = 0.035), GLS (*r* = −0.429; *P* = 0.030) and WBC (*r* = 0.321; *P* = 0.046). In addition, the percentage reduction of ICAM-1 at 12 months was associated with the corresponding changes of perfused boundary region (*r* = −0.643; *P* = 0.045) and %GLS (*r* = −0.500; *P* = 0.029). The percentage change of WBC at 12 months correlated with the percentage change of %GLS (*r* = −0.750; *P* = 0.020).

## Discussion

### Key findings

Our key findings indicate that EMPA treatment either before or after AMI in mice decreased no-reflow, inflammatory cell infiltration, and infarct size, and improved cardiac function. This study reveals that AMI induces profound transcriptomic alterations in ECs and fibroblasts 2 hours after reperfusion, while the effect of AMI on cardiomyocytes is minimal. EMPA pretreatment reverses the AMI-induced EC transcriptome alterations, indicating that these changes could account for the observed protection against microvascular injury or endothelial damage. For the first time, we present that the EMPA intervention retains its cardioprotective signal, even if it is administered at 60 minutes after reperfusion, with a mechanism that is related to the preservation of endothelial integrity and reduction of the infiltration of inflammatory cells in the infarcted zone of myocardium. In this study, we provide proof-of-concept clinical evidence for endothelial and cardiovascular parameters in patients with type 2 diabetes who received EMPA within a short period after AMI to facilitate future larger-scale studies. We observed endothelial glycocalyx protection, improved flow-mediated dilation of the brachial artery, and %GLS alleviation in STEMI patients treated with EMPA as well as improved circulating endothelial-related markers.

### EMPA treatment before or after AMI alleviates microvascular injury

Microvascular injury is characterized by dysfunction and damage of the myocardial microvasculature with a no-reflow phenomenon within the infarct zone.^30^ Microvascular injury has been shown to be an independent predictor of LV dysfunction and poor prognostic outcome after AMI, but we have yet to discover protective therapies to alleviate this deleterious phenomenon. The pathophysiologic mechanisms responsible for microvascular injury remain ill-defined and include enhanced vascular permeability, leucocyte adherence and transmigration, and capillary obstruction by erythrocytes and platelet-leucocyte aggregates.^30^ Proof-of-concept for the direct role of the endothelium in microvascular injury has been suggested through the use of imatinib^20^ and recombinant human angiopoietin-like 4.^31^ The effect of SGLT-2Is on the endothelium and microvascular injury has not been established.

As microvascular injury arises as a target for cardioprotection with future translational potential, the present study highlights the use of EMPA before and after AMI as a treatment to confer microvascular salvage. Our results regarding the pretreatment with EMPA agree with a recent study in pigs that demonstrated the efficacy of EMPA pretreatment in reducing infarct size and microvascular injury as well as preserving LV systolic function.^32^ Moreover, pretreatment with EMPA has been previously described to confer protection against microvascular injury via pathways related to mitophagy.^33^ Importantly, we now show that EMPA given after AMI is still protective. In parallel, our finding indicates the potential use of the drug in a translational setting of AMI with profound microvascular injury. The effect of SGLT-2Is on the endothelium and microvascular injury has not been established. The direct effects of EMPA on cardiomyocytes,^34^ ECs,^14^^,^^15^^,^^35^ and fibroblasts^36^^,^^37^ have shed light on the complexity of EMPA-mediated protection against cell death, but cell-specific alterations in vivo have not been previously addressed.

### Transcriptomic alterations after AMI in cardiac subpopulations and EMPA’s effects

This study provides a high-throughput and unbiased gene expression analysis of 3 cell populations of the ischemic cardiac tissue at 2 hours after reperfusion, which allowed us to identify which cell populations are affected by AMI and which are restored by EMPA pretreatment. Our results demonstrate that by using FDR <0.05, only 7 genes were differentially expressed between Sham and AMI groups in cardiomyocytes and nearly 600 genes were differentially expressed in ECs and fibroblasts. To the best of our knowledge, there are no RNA sequencing data at 2 hours after reperfusion in cardiomyocytes to compare with our data set. One interpretation of our finding would be that cardiomyocyte damage occurs within ischemia and the first minutes of reperfusion, which typically intensifies the morphologic features of cardiomyocyte necrosis.^38^ Therefore, cardiomyocyte transcriptomic alterations during late reperfusion are not profound because the damage has already occurred. Another interpretation of our finding lies with cardiomyocyte gene expression heterogeneity. Recently, single-cell transcriptomics revealed the transcriptional diversity within the cardiomyocyte population after AMI, which affects the variability. In that study, for example, only 1 distinct subpopulation of cardiomyocytes was characterized by the overrepresentation of stress-related hypertrophy genes.^39^ Further single-cell studies in the future could decipher the time course of transcriptomic and proteomic alterations in cardiomyocyte subsets after AMI.

Regarding the effect of EMPA pretreatment on the cardiomyocyte transcriptome, we revealed several transcriptomic changes related to mitochondrial metabolism pathways at 2 hours after reperfusion (but with FDR <0.10) that could partially explain the mechanism of EMPA-mediated cardioprotection. Because cardiac SGLT-2 expression is negligible, numerous potential mechanisms have been proposed for the direct cardiomyocyte and mitochondrial effects of EMPA, including the inhibition of cardiac Na^+^/H^+^ exchanger 1.^40^ The results of the transcriptomic analysis in cardiomyocytes are in agreement with our previous report that EMPA pretreatment increased complex I and II respiration at oxidative phosphorylation state in as early as the 10th minute of reperfusion in cardiac fibers.^16^ In the same study, dapagliflozin pretreatment led to infarct size reduction without altering mitochondrial respiration,^16^ and therefore whether the effect of SGLT-2Is on mitochondria function is causal for their infarct size–limiting properties remains to be determined in future studies.

In the present study, the EC transcriptome after AMI was examined as early as 2 hours after reperfusion, and profound gene expression alterations were identified related to cell adhesion and ECM composition. Very recently, the transcriptome of ECs in response to AMI was evaluated at several time points of reperfusion and the authors highlighted not only that ECs are highly compartmentalized in a temporal fashion, but also that AMI-related injury induces long-term transcriptomic alterations. Notably, genes for proteins involved in ECM degradation and remodeling, such as MMPs and TIMP-1 were identified as differentially expressed,^17^ in agreement with our findings.

Our data support the EC transcriptome as a potential target of cardioprotection by EMPA. A recent study demonstrated that dapagliflozin, when given 4 weeks before and 3 days after permanent coronary ligation, reduces cardiac damage via inhibition of macrophage activation in the area at risk even in the absence of SGLT-2.^41^ Although that single-cell analysis was performed in a nonhuman animal model without reperfusion, it revealed the role of macrophages and matrix remodeling in SGLT-2I’s cardioprotection^41^ in addition to the direct effect of EC protection reported in the present study. The effect of EMPA on the EC transcriptome in vivo is further supported by in vitro data by our group and others regarding the direct protective effect of EMPA on EC survival,^14^^,^^15^ which were recently summarized.^42^

### Mechanistic insights of EMPA’s cardioprotective effects at 2 hours and 48 hours after reperfusion

Ιncreased phosphorylation (Y705) of STAT-3 early in reperfusion has been proposed as one of the most robust cardioprotective signals causally associated with infarct size reduction.^43^ Numerous reports have demonstrated that cardioprotective interventions such as ischemic preconditioning, postconditioning, and remote ischemic conditioning activate the noncanonic function of STAT-3 as part of the survival activating factor enhancement pathway leading to infarct size reduction in a STAT-3–dependent manner.^43^, ^44^, ^45^, ^46^, ^47^ Our previous studies suggest that EMPA pretreatment increases the phosphorylation and activation p-(Y705)STAT-3^14^, ^15^, ^16^ and FGF-2 expression^16^ at the 10th minute of reperfusion and that the infarct size–limiting effect of EMPA pretreatment is STAT-3 dependent (identified by means of pharmacologic inhibition with STATTIC).^16^ We have also demonstrated that chronic administration of EMPA for 6 weeks, in exactly the same experimental protocol as the one applied in the present study, did not alter p-(Y705)STAT-3 2 hours after reperfusion.^14^ In the present study, pre-AMI EMPA tended to increase p-(Y705)STAT-3 without being significantly altered 2 hours after reperfusion, in agreement with our previous report.^14^ The temporal variation of STAT-3 regulation within the first hours of reperfusion with and without cardioprotective interventions has not been fully elucidated. We found that EMPA treatment before and after AMI significantly reduced STAT-3 phosphorylation at 48 hours after reperfusion compared with the control group. Sustained activation of STAT-3 after AMI has been reported to contribute to inflammatory processes and cardiac remodeling, and this activation is probably downstream of interleukin-6 via the gp-130 receptor^48^ whereas inhibition of JAK2/STAT-3 pathway attenuated cardiac hypertrophy.^49^ STAT-3 regulation may affect inflammation during AMI and very recently, the protective role of STAT-3 inhibition was described in a murine model of 30 minutes of ischemia and 24 hours of reperfusion.^50^ The pharmacologic STAT-3 inhibition by STATTIC reduced infarct size at 24 hours after reperfusion, which was associated with the reduction of anti-inflammatory macrophages in the area at risk via the interleukin-6/MCP-1/STAT-3 signaling, but the authors did not examine p-(Y705)STAT-3 phosphorylation.^50^ The STAT-3 activation at later phases of reperfusion has been mainly attributed to the invading macrophages in the heart tissue,^43^ and therefore the observed reduction in p-(Y705)STAT-3 by EMPA may be linked to the marked reduced inflammatory monocytes in the ischemic heart that we observed in this study rather than the signaling from cardiomyocytes.

Regarding the protein alterations 48 hours after reperfusion, our findings confirmed the transcriptomic changes and indicated that both pre-AMI and post-AMI EMPA significantly reduce ICAM-1 and MMP-2 protein levels which are consistent with reduced leukocyte adherence and improved ECM remodeling.^51^ These protein findings are consistent with the reduced inflammatory cell infiltration in the myocardium and the histologically conferred coronary endothelium integrity and allows us to connect 2 previously stated hypotheses regarding the effect of EMPA on ECs^14^^,^^32^ and on inflammation.^52^ Another point of connection among our findings is that increased p-(Y705)STAT-3 phosphorylation promotes the expression of MMP-2 by the direct binding of STAT-3 to the MMP-2 promoter, leading to a feedback loop that contributes to an uncontrolled inflammatory response.^53^, ^54^, ^55^ EMPA treatment before and after AMI reduced p-(Y705)STAT-3 phosphorylation and concomitantly the expression of MMP-2 and inflammatory cell infiltration at 48 hours of reperfusion, suggesting the possible anti-inflammatory effect of EMPA at later phases of reperfusion.

We are the first to report a therapeutic regimen that confers cardioprotection by EMPA treatment after AMI in an experimental model of 30 minutes of ischemia and 48 hours of reperfusion. This finding challenges the time frame for the application of therapeutic interventions against AMI-related cardiac injury. Cardiomyocyte cell death occurs in the first minutes of reperfusion, as hypercontracture appears to be the main cause of cardiomyocyte necrosis.^56^^,^^57^ For that reason, the notion that cardioprotective interventions must be applied as early as possible within the first few minutes of reperfusion to be effective has been supported. That notion is challenged by the fact that following the early reperfusion phase, vascular failure and microvascular injury contribute or exacerbate cardiac damage.^4^ In addition, cardioprotective interventions may fail to reach severely ischemic myocardium with little or no collateral flow, suggesting that the understanding of the sequence of pathologic events has significant implications for translating cardioprotection.^58^ The findings of the present study show that EMPA given 1 hour after reperfusion and once daily for 48 hours results in cardioprotection marked by infarct size, microvascular injury, and inflammation reduction, suggesting that the window for application of cardioprotective interventions lies within the first hours or even days of reperfusion.

### EMPA treatment after STEMI improves endothelial dysfunction, the perfused boundary region of microvessels, and %GLS in diabetic patients on top of metformin

We provide proof-of-concept clinical data regarding the impact of EMPA on the vasculature after STEMI in diabetic patients on top of metformin compared with insulin. EMPA treatment after STEMI reduces the perfused boundary region, suggesting endothelial glycocalyx protection. Although it was measured in the periphery, it could be linked to microvascular homeostasis and coronary microcirculation.^59^ Patients receiving EMPA after AMI did not experience exacerbation of pulse-wave velocity as early as the 4th month of treatment, providing evidence for reduced arterial stiffness and vascular resistance, in agreement with previous reports for SGLT-2I treatment with unspecified AMI incidence.^60^

Flow-mediated dilation is a measure of endothelial dysfunction in the periphery and correlates with coronary artery endothelial function as an independent predictor of cardiovascular outcomes.^61^ EMPA treatment improved flow-mediated dilation in STEMI patients with diabetes.

Finally, patients treated with EMPA had an improved %GLS at 4- and 12-month follow-up, in agreement with the in vivo data that post-AMI EMPA improves the %GLS. The protective effects of EMPA on cardiovascular function were observed on top of metformin, whereas these effects were not observed in patients treated with the combination of insulin with metformin. Therefore, the mechanism of vascular protection (in the periphery) by EMPA could be distinct from metformin^62^ and insulin signaling.^63^

In terms of endothelial integrity, we selected representative EC-related circulating markers, such as thrombomodulin and soluble ICAM-1. Inflammatory factors and neutrophil infiltration, directly or indirectly, lead to vascular EC damage and the release of a large amount of thrombomodulin,^64^ and ICAM-1 serves as a marker of endothelial damage and inflammation.^65^^,^^66^ EMPA-treated STEMI patients exhibit lower thrombomodulin and ICAM-1 levels, which indicate endothelial protection after STEMI but also reduced microvascular complications due to diabetes.^66^ In support of our findings, EMPA-treated patients with diabetes had decreased ICAM-1 circulating levels 24 weeks (6 months) after treatment initiation.^67^ The extent of microvascular injury determines the levels of circulating neutrophils, monocytes, and white blood cells.^68^^,^^69^ Patients receiving EMPA exhibited significantly lower WBC, possibly indicating less microvascular injury system damage after AMI.

Recently, Udell et al^9^ demonstrated that EMPA administration after AMI did not reduce the composite endpoint of hospitalization for heart failure or death from any cause. Similarly, treatment with dapagliflozin after AMI in patients without diabetes or heart failure, though it had beneficial effect on cardiometabolic outcomes, had no impact on the composite of cardiovascular death or hospitalization for heart failure.^10^^,^^11^ According to the authors, in the early phase of an AMI, several mechanisms may not be amenable to modification with SGLT-2 inhibition, including cardiac causes, such as stent thrombosis and recurrent myocardial infarction, and noncardiac causes during the first 30 days after AMI, contributing to mortality.^9^ Specific populations may benefit from EMPA treatment after AMI. In a subgroup analysis of EMPACT-MI (Effect of Empagliflozin on Hospitalization for Heart Failure and Mortality in Patients With Acute Myocardial Infarction; NCT04509674), EMPA given after AMI reduced the time to first hospitalization and total number of heart failure hospitalizations in patients with LV dysfunction or congestion, suggesting that patients at high risk of LV dysfunction could benefit from EMPA treatment.^10^ Pre-existing coronary microvascular dysfunction is regarded as an independent predictor of adverse cardiac events,^70^ and the detrimental role of endothelial dysfunction in AMI and the development of heart failure^65^^,^^71^ is gradually gaining interest as a therapeutic target, especially in patients with well known risk factors (eg, diabetes, hyperlipidemia, hypertension, etc).^70^^,^^72^ The present study suggests that EMPA administration in diabetic patients after AMI improves cardiovascular function, indicating that specific populations such as diabetic patients may benefit from SGLT-2Is in AMI. Our preclinical evidence suggests that patients with microvascular injury and no-reflow could benefit from EMPA treatment after AMI and could be considered as a target population to further investigate EMPA-mediated cardioprotection.

### Study limitations

Only male mice were used in this study, and sex may stand as a limitation to the interpretation of the murine study results. However, infarct size and no-reflow do not differ between male and female Göttingen minipigs,^73^ suggesting that our findings may be generalizable to both sexes. Our study includes a small cohort of patients, so we cannot determine causal associations with clinical outcomes or identify whether microvascular injury predicts which patients are most likely to benefit from EMPA after AMI. For the clinical study, patients who were referred to the cardiometabolic outpatient clinic, were randomly assigned to EMPA according to the applicable guidelines of that period. Therefore, EMPA administration was not performed immediately after reperfusion as in the animal study, limiting the interpretation of the findings from bench to bedside and back. In addition, the subgroup analysis was based on the criterion that the initiation of the EMPA treatment was within 2 months after STEMI, which is a variable time frame. Future studies with cardiac magnetic resonance imaging definition of microvascular injury and coronary endothelial dysfunction would provide evidence for the benefit of EMPA after AMI. Male sex is overrepresented in our patient study, and therefore sex may stand as a limitation to generalizability.

## Conclusions

This study highlights that oral EMPA treatment targets the EC transcriptome and prevents microvascular injury, leading to reduced infiltration of inflammatory cells. Our data support the administration of EMPA after reperfusion as a potential translational strategy against microvascular injury. EMPA attenuates damage to the coronary microvasculature, reduces infarct size, and improves global cardiac function in a murine model of AMI. At 48 hours after reperfusion, EMPA treatment both before and after AMI reduces ICAM-1, MMP-2, and STAT-3 phosphorylation in parallel with the reduction of inflammatory cell infiltration in the heart. In addition, post-AMI oral treatment with EMPA improves endothelial glycocalyx and flow-mediated vasodilation in STEMI patients with diabetes. These data warrant future work to examine the effect of EMPA against coronary microvascular injury on a larger scale, providing perspectives for the implementation of SGLT-2 inhibitors in the setting of AMI.

## Data Availability

The data underlying this article will be shared on reasonable request to the corresponding author. The raw RNA-sequencing data are publicly available in GEO under the accession no. GSE255933.

Perspectives **COMPETENCY IN MEDICAL KNOWLEDGE:** Microvascular injury has emerged as a major determinant of heart failure and prognosis in patients with acute myocardial infarction. However, therapies proven to reduce microvascular injury are lacking. Empagliflozin preserves microvascular integrity and cardiac function in the setting of acute myocardial infarction, providing evidence for the administration of empagliflozin to alleviate microvascular injury.

**TRANSLATIONAL OUTLOOK 1:** Empagliflozin restores the cardiac endothelial cell transcriptome and gene expression related to adhesion molecules and matrix degradation and prevents microvascular injury, leading to reduced infiltration of inflammatory monocytes and neutrophils.

**TRANSLATIONAL OUTLOOK 2:** The administration of empagliflozin after acute myocardial infarction may confer protection to the vasculature and benefit populations with profound coronary microvascular injury manifestations, potentially indicated by circulating markers such as ICAM-1.

## Funding Support and Author Disclosures

The authors acknowledge support of this work by the project “The Greek Research Infrastructure for Personalised Medicine (pMedGR)” (MIS 5002802) under the Action “Reinforcement of the Research and Innovation Infrastructure,” funded by the Operational Programme “Competitiveness, Entrepreneurship and Innovation” (NSRF 2014-2020) and co-financed by Greece and the European Union (European Regional Development Fund). This study was partially supported by an investigator-initiated study from Boehringer Ingelheim to Dr Andreadou. Dr Pieper is an employee of Boehringer Ingelheim Pharma & Co; and provided the research grant to the research institute of Dr Andreadou related to this work. Dr Zuurbier has received a research grant from Boehringer Ingelheim. Dr Nijveldt has received research grants from Philips Volcano and Biotronik; and speaker fees from BMS, Pfizer, and Sanofi Genzyme. Dr van Royen has received research grants from Abbott, Philips, Medtronic, and Biotronik; and speaker fees from Abbott, Bayer, RainMed, and Microport. All other authors have reported that they have no relationships relevant to the contents of this paper to disclose.
