# Supplementary material for: Empagliflozin in Acute Myocardial Infarction Reduces No-Reflow and Preserves Cardiac Function by Preventing Endothelial Damage
Source: JACC Basic Transl Sci. 2024 Aug 30;10(1):43–61. doi: 10.1016/j.jacbts.2024.08.003 (PMC11830260; doi:10.1016/j.jacbts.2024.08.003)
Supplement: Supplemental Material [file mmc1.pdf]

SUPPLEMENTAL APPENDIX

Supplemental Methods

Methods are presented in logical order following the presentation of the results in the manuscript.

1. Reagents and pharmacological agents

Empagliflozin (EMPA) pure powder was kindly provided by Boehringer Ingelheim (BI 10773XX, Batch Number:1082812). For the implementation of the *in vivo* protocol, a fresh drug suspension was prepared, based on our previous experience on EMPA[1–3]. Briefly, EMPA powder was weighted and diluted in the corresponding volume of 5% DMSO in sterile nonpyrogenic normal saline and the suspension formed was administered to the animals by oral gavage (per os administration), as previously described[1–3].

All reagents used for the experiments were purchased from Sigma Aldrich (via Life Science Chemilab SA and Tech-Line Chemicals SA, Athens, Greece), while Collagenase II was purchased from ThermoFisher Scientific (Catalog #17101015) (via ANTISEL Medical Equipment & Supplies, Athens, Greece). The primers used for Real-Time Polymerase Chain Reaction (RT-PCR) and Polymerase Chain Reaction (PCR) were synthesized by Eurofins Genomics AT, GmbH.

2. Animal protocol details

Mice (n=6/cage) were housed in a specific pathogen-free facility in a temperature-controlled environment (20-25 °C), under 12-h light/dark cycle and received regular laboratory animal diet and water ad libitum. Surgical procedures and interventions were performed in compliance with the guidelines[4], i.e., mice randomization was performed in all experiments, surgical procedures and analysis of the experimental

data were performed in a blinded manner which was ensured by Castor EDC (v2022.4.1.3). Exclusion criteria were defined *a priori* as the absence of ST-elevation in electrocardiography and the rupture of the suture/vessel during the surgical procedure for AMI induction.

2.1 Sample size

In the first series of experiments, the aim was to detect the cell populations transcriptionally affected by acute myocardial infarction and EMPA treatment. Based on our previous experience with EMPA treatment in mice and proteomic studies[1,2] as well as recent reports for RNA-sequencing (RNA-seq) sample size estimation[5], we aimed for a total of n=4-6 biological replicates per cell population/ per group taking into account that different cell populations were analyzed. Every effort to minimize batch effect for the identification of differentially expressed genes (DEGs) was made. As such RNA library preparation and sequencing run were performed on the same time for all samples and randomization with cage mate controls was applied. N=2 preparations led to inadequate RNA quality/quantity for the experiment and were excluded *a priori* from analysis.

In the second series of experiments, the sample size was determined with power calculations *a priori* based on previously published values regarding the mRNA gene expression of *Vegb* in EMPA treated groups[1]. To detect the gene expression difference with $\alpha = 0.05$, power = 0.80, standard deviation (SD) = 1, at least n = 6 successful experiments per treatment group are necessary for the conduction of the *in vivo* experiments[1]. The power calculation for the second series of experiments is depicted in ***Supplemental Figure 1***. No exclusions or deaths are reported for the second series of experiments and therefore n=6 biological replicates are presented.

In the third series of experiments, the least number of animals n=3 per timepoint were included for the determination of EMPA's concentration in the murine plasma (total n=9) since previous extensive reports of pharmacokinetics have been published[6]. For

the standard curve, a total of n=3 animals were used as control (without administration of EMPA). No exclusions or deaths are reported for the third series of experiments and the results of n=3 animals per timepoint are presented.

The primary outcome of the present study is the determination of microvascular injury (MVI) and therefore for the sample size calculation of the fourth series of experiments we performed power calculations *a priori* based on previously published values[7]. To detect a relevant reduction in microvascular obstruction (MVO) with $\alpha = 0.05$, power = 0.80, SD = 6, at least n = 12 successful experiments per treatment group are necessary for the conduction of the *in vivo* experiments. The samples size calculation was based on previous report regarding the effect of EMPA on MVO in pigs (mean values 15.2, 7.2 and 10.3 with an SD=6). A dropout of 25% is taken into account according to previous reports[2,8] and therefore for groups Group 2- Control-AMI, Group 3- EMPA-Pre-AMI, Group 4- EMPA-Post-AMI, n=16-18 animals were allocated while for Group 1-Sham in which we expected no mortality rate and the absence of no-reflow phenomenon we allocated n=8 animals. The power calculation for the fourth series of experiments is depicted in ***Supplemental Figure 2***. In this experimental series, 8 deaths (mortality 13%) were reported due to intubation or surgical mishandling, while n=4 mice were excluded *a priori* from analysis (3 without ST-elevation and 1 with ruptured LAD) and the final group numbers were n=8 (Group 1), n=13 (Group 2), n=14 (Group 3) and n=13 (Group 4). The presence of two outlying values in the group of EMPA-Post-AMI were identified by GraphPad Prism analysis software, using the ROUT method and Q=1% for the measures of no-reflow to left ventricle and no-reflow to area at risk (***Figures 3C and 3D***).

In the fifth series of experiments, the sample size to detect a reduction in inflammatory cell infiltration by the treatment was determined based on previous reports both from our group[9] and others[10] where n=6-8 biological replicates were employed.

2.2 Animal care and monitoring

All the procedures were performed by experienced technicians/researchers. In this way the oral gavage procedure imposed the least stress possible on the animals. During the surgery of the ischemia/reperfusion the animals were completely anesthetized, and anesthesia was monitored regularly with toe pinch so that the animals do not experience any distress. Analgesia was also provided when the surgical induction of ischemia /reperfusion included recovery of the mice, in the fourth and fifth experimental series. For all experimental series the humane endpoints were predefined and written to the respective animal experimentation protocols for approval. These included the loss of body weight of 20%, body condition score (BCS) ≤2, respiratory distress after surgery and inability to reach food and water. The animals were monitored daily and none of the animals included in the protocol reached the humane endpoints.

3. Surgical procedures (acute myocardial infarction–2 h reperfusion)

The induction of acute myocardial infarction (AMI) was performed surgically. Mice (n=48) were anesthetized with an intraperitoneal injection (i.p.) of a combination of ketamine (100mg/kg), xylazine (20 mg/kg), and atropine (0.6 mg/kg). Anesthesia depth was monitored by the loss of pedal reflex. Body temperature was maintained at 37°C throughout the surgical procedure with the use of a heating pad. Tracheotomy was performed for artificial respiration via a rodent ventilator at a rate of 200 strokes/min and with tidal volume of 0.18 mL. After left-sided thoracotomy, the left anterior descending coronary artery (LAD) was ligated approximately 3-4 mm distal to its origin under the left atrium using a 6-0 silk suture and the heart was allowed to stabilize for 5 min. After the ischemic period, the ligature was released and allowed reperfusion of the myocardium for 2 h, as previously described[1–3]. Mice hearts were either used for RNA-seq analysis or RNA isolation as described below.

4. Cell sorting and RNA-sequencing

4.1. Heart digestion and cardiomyocyte isolation

To isolate the cell populations from the murine heart, we applied a protocol that provides an efficient method to digest the heart and recover a high number of viable cells[11,12]. To isolate sufficient quantity of high-quality RNA, we pooled together 2 hearts per sample and performed 4-5 independent experiments per cell population per group. Therefore, the cardiomyocyte, the fibroblast and the endothelial cell expression profiles at 2 hours or reperfusion represent average from 8 to 10 mice. This strategy has been recently described to strengthen the statistical power of experimental results and has resulted in the identification of various gene expression alterations in endothelial cells in the context of AMI[13].

In these experiments, labware and buffers used were sterilized and produced with PCR-grade water. At the end of the reperfusion, an intraperitoneal dose of 0.5 mL of heparin diluted in normal saline to 100IU/mL was administered to the animal, to prevent the formation of blood clots during heart perfusion and digestion. After 10 min, the heart was excised. Then, a stainless-steel cannula was cannulated into the aorta, under the magnification of a stereoscope. The aorta was then tied to the cannula using a 6-0 silk thread and was placed onto the Langendorff perfusion system.

The heart was initially perfused with the perfusion buffer (***Supplemental Table 1***) for 4 min at 4 mL/min, to flush blood from its vasculature and then perfusion was switched to the digestion buffer (***Supplemental Table 2***) for 3 min at 4 mL/min to perform heart digestion (collagenase type II). Then, 15 μL of 100 mM $CaCl_2$ was added to the myocyte digestion buffer reservoir and digestion was continued for another 8 min. After heart digestion, the ischemic part below the suture was cut from the heart, put into a petri dish with 2.5 mL of myocyte digestion buffer and was transferred into a cell culture laminar flow. Right after, it was gently torn apart using a pair of sharp tweezers and pipetted up and down, until a cell suspension was created. Then, 5 mL of myocyte

stopping buffer (***Supplemental Table 3***) was added to stop collagenase II activity and prevent over-digestion. The cell suspension was transferred into a 15 mL sterile polypropylene conical tube and centrifuged for 3 min at 20*g* to precipitate rod-shaped cardiomyocytes (CMs). The supernatant, which mainly contained the non-cardiomyocyte populations [leukocytes, fibroblasts; (FBs), endothelial cells; (ECs), etc.] and cell debris was collected into a 15 mL sterile conical falcon tube and remained on ice until FACS analysis while the pellet, mainly comprising of CMs, was resuspended in 10 mL of myocyte stopping buffer.

Then, 100uL of CMs from two different mice were mixed as one biological replicate and were collected in an RNAase-free Eppendorf tube. Cells were lysed with the RL-cell lysis buffer obtained in the single cell RNA purification kit (Norgen Biotek, Product #51800) and the mixture was vortexed vigorously to perform cell lysis, which is the first step towards RNA isolation.

4.2. Cell-sorting of FBs and ECs using fluorescense-activated cell sorting (FACS)

The supernatant which contained the non-CM cells was centrifuged (5 min, 300*g*, 4°C) and the supernatant was discarded. At this time point, 4 mL of the Red Blood Cell lysis buffer made with RNA-ase free water was added to the cell pellet and mixed. The cell suspension was then incubated for 10 min at room temperature, in the dark, to lyse the red blood cells and abolish their interference during the flow cytometric analysis. After red blood cell lysis, 8 mL of PBS containing 2% fetal bovine serum (PBS/2% FBS) were added, and the subsequent mixture was passed through a sterile 40 um cell strainer to obtain a uniform single-cell suspension and prepare our sample for the cytometric analysis. The cell suspension was centrifuged immediately (5 min, 300*g*, 4°C) and the supernatant was decanted. Right after, 200 μL of PBS/2% FBS were added to the pellet and the suspension formed was transferred into a 1.5 mL Eppendorf. To prevent non-specific binding of the fluorescent antibodies, cells were

firstly incubated (15 min, 4°C, light-protected) with 1 μL of anti-CD16/32 Fc block, with mild handshake every 5 minutes (1:200). Blocking was followed by cell incubation (30 min, 4°C, light-protected with mild hand shake every 10 min) with the three fluorescent antibodies. Optimal antibody concentrations were determined by titration and were 1:250 for anti-CD45-FITC, 1:100 for anti-CD31-PE and 1:100 for anti-CD90-APC-Cy7. The antibodies used for cell shorting were purchased from BD Pharmigen, San Diego, CA, USA (1. Purified Rat Anti-mouse CD16/CD32 Fc Block Catalog #553142 2. FITC Mouse Anti-Mouse CD45.1 Catalog #561871, 3. APC-Cy7 Rat Anti-Mouse CD90.2 Catalog #561641, 4. PE Rat Anti-Mouse CD31 Catalog #561073). Afterwards, the cell suspension was centrifuged (300*g*, 5 min, 4°C), to wash off the unbounded antibody, and the supernatant was discarded. Finally, the cell pellet was resuspended in 2 mL PBS/2% FBS and the suspension formed was transferred into a 5-mL sterile RNAase-free polypropylene tube (Falcon Round-Bottom Polypropylene Tubes, STEMCELL Technologies, Catalog # 100-0089).

The FACS Melody Cell Sorter (BD Biosciences, San Diego, CA, USA) and the BD FACS Chorus Software (BD, San Diego, CA, USA) were employed to detect and sort FBs and ECs from the cell suspension. The construction of our gating strategy was performed as follows: Firstly, a forward scatter height (FSC-H) versus forward scatter area (FSC-A) plot was designed to exclude doublets, as these can significantly affect our analysis. Further doublet exclusion was performed using the side scatter height (SSC-H) versus side scatter area (SSC-A) plot, since this type of gating increases sensitivity of doublet exclusion. Then, an SSC-A versus FSC-A plot was created. Given the fact that FSC indicates cell size, whereas SSC relates to the complexity/granularity of the cell, we chose events with high FSC (to exclude cell debris and erythrocytes, that are much smaller than most other murine cells) and low SSC (to further exclude doublets) (P3 population). We further analyzed the P3 population, using the anti-CD45-FITC, to exclude $CD45^+$ cells. The $CD45^-$ population (P4 events) was further analyzed using anti-CD31-PE and endothelial cells ($CD31^+$, P5 events) were identified. $CD31^-$

cells were further divided to CD90$^+$ cells that comprise of FBs (P7 events) and CD90$^-$ cells that can be identified as other cell populations (***Supplemental Figure 3***).

Samples were sorted into sterilized RNAase-free polypropylene tubes, each one containing 2 mL of the RL-Lysis buffer (provided within the Norgen single-cell RNA isolation kit), inserted into the cell collection chamber. The cells were sorted immediately into the RL-Buffer to eliminate sticking on the tube's walls, increase the collected RNA quantity and prevent RNA degradation, as the RL-Buffer contains different RNA-ase inhibitors that will eventually preserve RNA integrity, increasing RNA quality. After sorting the whole sample, the two tubes containing our cells were vortex vigorously to promote cell lysis and RNA extraction. The formed cell lysate was subsequently used for RNA purification and isolation.

4.3. RNA isolation for RNA-sequencing

To obtain high quality RNA for RNA-seq, we used the single-cell RNA purification kit that was purchased from Norgen Biotek Corporation (Catalog #51800). Following the manufacturers' instructions, the cell lysate that was formed upon the mixture of each cell population with the corresponding volume of RL-Buffer was collected as described above. Then, a corresponding volume of ethanol was added into the cell lysate (200 μL ethanol 96-100% for every 350 μL of RL-Buffer) and the mixture was vortexed vigorously for 10 sec. Right after, a Single Cell RNA Spin Column was assembled with one of the provided collection tubes and the mixture of the lysate with ethanol was applied onto the column and centrifuged (1 min, 3.500*g*, 25°C) for binding RNA to the column. Then, the flow-through was discarded, the spin column was reassembled with its collection tube and the previous steps were repeated until the total volume of the cell lysate/ethanol mixture was passed through the column. Subsequently, 400uL of Wash Solution A was added to the column and was centrifuged (1 min, 14.000*g*, 25°C) to wash the column. The washing step was repeated twice, and the spin column was centrifuged again (2 min, 14.000*g*, 25°C) to thoroughly dry the resin. The RNA bound

on it, was placed into a 1.7 mL RNA-ase free Elution tube, provided with the kit. Finally, 12 μL of Elution Solution A was added to the column and then centrifuged (1 min, 200*g*, 25°C) to allow the Elution Solution A to enter the column and dilute the RNA. A second centrifugation (1 min, 14.000*g*, 25°C) was then performed to elute the RNA from the column. The eluent was reintroduced into the column and the previous two centrifugations were repeated to totally elute the bound RNA from the column. Finally, the purified RNA samples were stored at -80°C until use for downstream application.

4.4. RNA sequencing

The RNA samples were transferred to the RNA-sequencing facility, “pMedGR” Research Unit for the implementation of Next Generation Sequencing (NGS). 38 total RNA samples were used for library construction. RNA concentration was determined using a Qubit-4 Fluorometer (Invitrogen) and the Qubit RNA HS Assay Kit (Invitrogen). RNA quality was determined on an Agilent 2100 Bioanalyzer (Agilent) using the Agilent RNA 6000 Nano Kit (Agilent) and protocol. For library preparation, the QuantSeq 3‘mRNA-Seq Library Prep Kit for Illumina (FWD) (Lexogen) was used according to manufacturer’s instructions. Briefly, up to 500 ng of RNA from each sample were used for first strand synthesis, followed by RNA template removal. Second strand synthesis was initiated by random primers containing compatible linker sequences at its 5’ end. In-line barcodes were introduced during second strand synthesis followed by magnetic bead-based purification; the resulting libraries were amplified for up to 15 cycles and re-purified. Quality and quantity of libraries were assessed on an Agilent 2100 Bioanalyzer using the High Sensitivity DNA Kit reagents and protocol (Agilent). The quantified libraries were pooled at a final concentration of 2 nM and sequenced on a NextSeq 550 System (Illumina), according to manufacturer’s instructions.

4.5. Bioinformatic analysis

Adapter content of RNA-seq samples was estimated by employing Minion v.15-065 from the EBI Kraken tools (https://www.ebi.ac.uk/research/enright/software/kraken). Quality control of raw and pre-processed sequencing files was conducted using FastQC Suite (www.bioinformatics.babraham.ac.uk/projects/fastqc/), followed by removal of low-quality reads (i.e. -q 20) using cutadapt[14] (v2.8) and curation of the resulting length distribution of reads. RNA alignment and quantification was performed by utilizing Salmon[15] against GRCm39 genome assembly and Ensembl[16] v106 genomic annotation.

Analyses detailed below were performed using the programming language R (v4.0.3). Assessment of the clearance of the different cell populations was achieved by performing marker-based decomposition of bulk RNA-seq expression using the R package BisqueRNA[17] and marker genes for each cell type from PanglaoDB[18]. Heart gene markers for Cardiomyocytes, Vasculature for Endothelial Cells and Connective Tissue for Sorted Fibroblasts. Then, the discrimination of the cell populations was visually inspected through Uniform Manifold Approximation and Projection (UMAP) analysis. Differential expression analysis was performed using the R package edgeR[19], separately for each cell population (CMs, ECs, FBs). Briefly, lowly-expressed genes were filtered out by using the edgeR function filterByExpr with default parameters. Then, normalization factors of sample libraries were calculated using the Trimmed Mean of M-values (TMM)[20] method, followed by estimation of the negative binomial dispersion and application of generalized linear models with quasi-likelihood tests (edgeR::glmQLFit function) for the statistical analyses, robustifying the model in both cases against hypervariable genes. False Discovery Rate (FDR) was controlled by applying the Benjamini-Hochberg method[21]. Pathway enrichment analysis was conducted using the kegga function from the R package limma, utilizing only statistically significant differentially expressed genes for each contrast and pathway resource annotations from Reactome[22]. Graphs were created using the R packages

EnhancedVolcano (volcano plots), PCA tools[23] (Prinical Component Analysis plots) and ggplot2[24] (UMAP, heatmaps) (https://ggplot2.tidyverse.org.)

To ensure effective discrimination of the cell population transcriptome, we employed a decomposition package used for single cell RNA sequencing taking into account the characteristic gene markers per cell type and sample namely Myl2, Myl4, Myh6 and Myh7 for CMs, Kdr, Fabp4 and Vwf for ECs, Col1a1, Dcn, and Lum for FBs. The decomposition UMAP indicates that the samples are discriminated into the correct cell populations (***Supplemental Figure 5***). We manually searched for characteristic gene expression markers in the different cell populations to ensure their purity. Interestingly, in the FBs population we indicated a remarkable overexpression of the markers CD29 Integrin 1beta, Vimentin, CD90 which also advocate for FBs purity and in ECs we detected the overexpression of the markers CD31+/PECAM 1, eNOS, CD144/VE-Cadherin which ensure ECs purity. Finally, in CMs we observed the overrepresentation of the markers: NKX2-5, Cardiac Muscle Troponin T2, Cardiac Muscle Troponin I, Myh6 Myocin which advocate for CMs purity.

5. Validation of RNA-sequencing results

5.1. RT PCR

For the isolation of RNA from the ischemic part, the snap-frozen murine hearts were pulverized and extracted by the standardized Trizol protocol[1]. RT-PCR was performed with the CFX96 Real-Time PCR Detection System (Bio-Rad, Munich, Germany). Isolated RNA was reverse transcribed to cDNA using FastGene Scriptase II cDNA Kit (NIPPOIN Genetics EUROPE, #LS63). Primer pairs were designed (Primer-Blast, NCBI, NIH) and were synthesized by Eurofins Genomics AT, GmbH. The primers were used to detect the mRNA expression of genes are depicted in the ***Supplemental Table 4*** and Gapdh gene was used as endogenous control. The standardized EvaGreen® Dye method (Biotinium) was employed according to the manufacturer's

instructions for RT- PCR experiments. To investigate changes in gene expression, we performed RT-PCR and the comparative delta CT method was used for relative mRNA quantification. Gene expression was normalized to the endogenous control (*Gapdh*), and the expression of the target gene mRNA of each sample was expressed relative to that of the control.

5.2. Selection of genes

We manually evaluated the 150 genes and via literature search we selected some genes that are affected the most and could be related to the AMI damage. Among these, we chose to evaluate the gene expression of: 1) growth factor genes and regulators related to ECs survival namely insulin-like growth factor 1 (*Igf)* and epidermal growth factor receptor *(Egfr).* We also examined the effect of the deregulated but not significant genes of vascular endothelial growth factor a and b (*Vegf-a Vegf-b),* 2) genes related to cell adhesion and ECs migration namely *Syndecan-2,* Superoxide dismutase-3 (Sod-*3)* and *Rab7b* and we also examined the effect of the deregulated but not significant gene of Intercellular adhesion molecule-1 (*Icam)* and 3) genes related to collagen degradation and extracellular matrix namely Matrix metalloproteinases 2, 14 and 23 (*Mmp-2 Mmp-14, Mmp-23),* and the deregulated but not significant genes of Tissue inhibitors of metalloproteinases -1 and -2 (*Timp-1*,*Timp-2).*

6. Liquid chromatography-mass spectrometry for quantification of empagliflozin in murine plasma

The quantification of EMPA in plasma samples was performed via a novel Liquid chromatography-tandem mass spectrometry (LC-MS/MS) method, following protein precipitation for sample pretreatment. In brief, 80 μL of plasma samples were mixed with 250 μL of acetonitrile, containing 50 ng/mL of internal standard (dapagliflozin). The 1.5 mL eppendorf tubes with the mixture were vortexed for 5 min and then centrifuged

for 10 min at 10,000 rpm. Aliquots of 210 μL of the supernatant were transferred to the respective wells of a 96-well plate for direct LC-MS/MS analysis.

EMPA analysis was conducted on a Sciex API 4000Qtrap mass spectrometer (Antisel, Greece) in negative electrospray mode. EMPA and internal standard were detected as acetate adducts ($[M + CH_3COO]^-$) by utilizing the precursor → product ion transition (multiple reaction monitoring transitions), performed at m/z 509.2 → 371.1 for EMPA and 467.2 → 329.1 for dapagliflozin, respectively. The chromatographic separation was performed under isocratic elution on a YMC octyl column, 3 μm, 120 Å, (50 × 4.0 mm i.d.) (Schermbeck, Germany) by a Shimadzu LC system (Asteriadis, Greece). The mobile phase consisted of acetonitrile: ammonium acetate 30 mM with pH adjusted to 5.5 with acetic acid, 70:30 (v/v) and was provided at a flow rate of 0.7 mL/min. The injection volume was 40 μL, the autosampler temperature was set at 10 °C and the total run time was set for 2.5 min and under these conditions EMPA and dapagliflozin were eluted at 0.93 and 0.98 min, respectively.

Unknown samples were quantifed by a calibration curve containing 8 standards, ranging from 5.0 to 2,000 ng/mL, using peak area ratios of EMPA to internal standard for regression analysis. A linear regression model (y=0.0105x+1.41, r=0.9986) was obtained via Analyst v1.6 software, using 1/x2 as weighting factor. The current method was validated according to FDA bioanalytical method validation guidelines and all criteria were within the acceptance limits. Quantification results are presented in ***Supplemental Table 5***.

7. Surgical procedures (acute myocardial infarction–48 h reperfusion)

Mice were anesthetized with a mixture of midazolam (7.5 mg/kg), medetomidine (0.75 mg/kg) and fentanyl (0.075 mg/kg) i.p. and were hydrated with 1mL of normal saline. Mice were subjected to endotracheal intubation, left-side thoracotomy and LAD ligation for 30 min followed by reperfusion. The animal's chest was closed and the effect of anesthesia was reversed using a mixture of atipamezole (2.5mg/kg) and flumazenil

(0.5mg/kg). Buprenorphine 0.5 mg/kg was administered right after surgery and thereafter every 12 h for pain relief. Attention was given on the presence of eye moisturizing ointment each mouse during the surgery[25].

8. CMR protocols

After 48 h of reperfusion, a tail vein cannula was placed for injection of the contrast agent. General anesthesia was induced by inhalation of 4% isoflurane, followed by inhalation of 1-2% isoflurane in a mixture of O_2/air = 1:2 via a nose cone.

Mice (n = 52) were transported to a 11.7 T BioSpec Avance III small animal MR system (Bruker BioSpin, Ettlingen, Germany), equipped with a transmit volume coil and a receive surface coil (10 mm diameter). Mice were placed in supine position and were connected to a 3-lead ECG with respiration sensor. Respiration rate was maintained at 60–80 beats per minute. Core body temperature was monitored using a fiber-optic temperature probe and maintained at 37°C using a warm waterbed.

The cine images were acquired using a 2D fast low angle shot sequence (typical voxel size 0.15x0.15x0.80 mm^3 and 30 phases per cardiac cycle) with retrospective electrocardiographic gating. The heart was imaged in four orientations: short axis (SAX), 2 chamber long axis (2CH), 3 chamber long axis (3CH), and 4 chamber long axis (4CH). Consecutive cine SAX images were acquired from base to apex. To study infarct size, an extracellular gadolinium-based contrast agent (Dotarem, Guerbet, Roissy, France) was injected via the tail vein cannula (0.25 mmol/kg) or, in case the tail vein cannula could not be positioned, intraperitoneally (1.00 mmol/kg). Late gadolinium enhancement (LGE) imaging was performed 10 min after contrast injection via tail vein cannula or 20 min after i.p. contrast injection, using a T1-weighted sequence (typical voxel size 0.10×0.10×0.80 mm^3). Consecutive LGE SAX images were acquired from base to apex to measure infarct size in identical slice positions as the cine SAX slices. CMR scanning parameters are depicted in ***Supplemental Table 7***.

8.1. CMR analysis

All CMR analyses were performed blinded to treatment allocation using a commercially available software (Medis, QMass 8.1 software, Medis Medical Imaging Systems, version 4.0.62.4, Leiden, The Netherlands). Epicardial and endocardial contours were manually drawn in left ventricular (LV) end-systolic and LV end-diastolic phase on the SAX cine images to measure LV mass and volumes and calculate left ventricular ejection fraction (LVEF). Infarcted myocardium was defined as the mean signal intensity exceeding at least five standard deviations from the mean signal intensity of remote myocardium and was manually adjusted afterwards when necessary. Total infarct size was calculated as infarct area multiplied by slice thickness and expressed as a percentage of LV mass (%LV).

8.2. CMR feature tracking analysis

Cardiac strain was measured on the cine images by means of feature tracking strain analysis (Medis, Qstrain software, Medis Medical Imaging Systems, version 4.3.6.2). Endocardial contours were manually drawn at the end-systolic and end-diastolic phase and contours were automatically tracked throughout the cardiac cycle. Left ventricular global longitudinal strain (GLS) was calculated as the average of strain measured on the 2-, 3-, and 4-CH long-axis cine images. Global circumferential strain (GCS) was measured as the average strain on the basal-, mid- and apical SAX cines.

9. Infarct size and no-reflow assessment

After the CMR scan, mice were removed from the CMR scanner under isoflurane anesthesia 1-2%. Anesthesia was increased to 2-3%, the chest of the mouse was surgically opened and the heart was exposed. A solution of 4% Thioflavin S (ThioS) in normal saline was injected directly in the apex of the heart at a rate of 20µL per 5 sec

for 40 sec. The heart was allowed to pump the ThioS for 2 min. At this timepoint, the murine hearts were gently excised, and the aorta was cannulated. The heart was perfused with Krebs Buffer and in this way the intravascular erythrocytes are washed and therefore the erythrocytes that are still present are either extravasated or obstructed. Afterwards, the suture was re-tightened and Evans Blue solution (1% in PBS) was slowly infused to delineate the ischemic from the normally perfused part of the myocardium. The hearts were immediately sliced in 1-2mm sections. The Evans blue and ThioS stainings were immediately imaged using a Nikon D3200 camera with Sigma 105mm F2.8 EX DG Macro OS lens. The Evans blue was captured in bright light while for ThioS the room lights were closed (complete dark) and the hearts were photographed under a 366nm UV lamp. Slices were photographed from both sides on a Kaiser RS 2 XA stand so that the distance from the camera and the heart slice remained the same. Then, the slices were incubated with Triphenyl-Tetrazolium Chloride Solution (TTC, 1% in PBS, pH=7.4) at 37°C for 10 min. Subsequently, the sections were photographed and the overall size of each slice, the area-at-risk, the no-reflow area and the infarcted area were determined using ImageJ software. Prior to measurements, the images of each slide were cropped using GIMP software and pasted as images with standard pixel numbers[1,2].

10. Histology

After the TTC and ThioS stainings, myocardial tissue slices were fixed in 4% buffered formalin solution for 24 h and embedded in paraffin wax for histopathological evaluation. 5 μm sections were obtained from each sample and routinely counterstained with hematoxylin and eosin (HE) for histological examination. The latter was performed under a light microscope (Nikon Eclipse 80i, Nikon Corp, Tokyo). Overview images were obtained with the Olympus EP50 digital camera on Olympus SZ61 stereoscope. The score for microvascular injury was assessed by blinded researcher as the percentage of the damaged vessels (disrupted or obstructed)

observed in the left ventricle. Score 0 was considered as equal or less than one damaged vessel, score 1 was considered as equal to or less than 25% of damaged vessels, score 2 as above 25% and equal to or less than 50% of damaged vessels and score 3 as more than 50% of the vessels observed being damaged.

11. Electron microscopy

From the mid-ventricular slices, small biopsies (1 mm^3) were taken from the center of the infarct area, based on negative ThioS staining. Biopsies were fixed in 2% (v/v) glutaraldehyde (Electron Microscopy Sciences (EMS)) in 0.1 M cacodylate buffer (EMS) at 4 ℃ for 24–48 h and post-fixed for 30 min in 1% osmium tetroxide (JEOL) and 1% potassium ferrocyanide (VWR) in 0.1 M Cacodylate buffer. After being washed in buffer, cells were dehydrated in an ascending series of aqueous ethanol and were subsequently transferred via a mixture of ethanol and Epon to pure Epon as embedding medium. Sections of 80 nm were cut and attached to a copper grid. Prior TEM imaging, the sections were contrasted for 45 minutes 1% uranyl acetate and for 7 minutes 3% Lead citrate (Ultrostain II, Leica) using an automatic contrasting instrument (AC20, Leica). Transmission electron microscopy images were recorded using a JEOL JEM 1400, at 60 kVThe number of erythrocytes per 1000μm^2 of area were counted.

12. Flow cytometry

A total of n=36 mice were used for this study and were annotated n=6 for the Sham group and n=10 per group for Control-AMI, EMPA-Pre-AMI and EMPA-Post-AMI. Three animals died during surgery and nine animals are presented. Spleen and blood analysis was performed in six animals per group. After 48 h of reperfusion the ischemic myocardium was collected and digested with collagenase type II (1 mg/mL; in Minimal Essential Medium for 30 min at 37°C. The cell suspension was passed through a 70µm strainer, lysed with red blood cell lysing solution for 3 min and centrifuged at 400*g* for

10min. After discarding the supernatant, the pellet was resuspended with cold PBS/2%FBS. The single cell suspension obtained from myocardial tissue was then evaluated for cell density and cell viability with Trypan-blue staining using a hemocytometer. The minimum cut-off point set for cell viability was 90% for further processing. Cells were then incubated with anti-CD16/32 Fc-block mAb (#553142, BD Pharmingen, San Diego, CA, USA) for 15min at 4°C to prevent unspecific binding and then stained with anti-Ly6G-PE (#551461, clone 1A8, 1:400,), anti-Ly6C-FITC (#553104, clone AL-21, 1:200,), anti-CD11b-APC (#553312, clone M1/70, 1:200), anti-CD19-PE-Cy7 (#552854, clone 1D3, 1:400), anti-CD3e-PerCP-Cy5.5 (#551163, clone 145-2C11, 1:200), anti-CD4-BV510 (#563106, clone RM4-5, 1:1000), anti-CD8a-APC-Cy7 (#557654, clone 53-6.7, 1:400) for 30min at 4°C. Stained cells were centrifuged at 300*g* for 5 min at 4°C, washed twice with PBS/2%FBS. The acquisition of all samples was performed on a BD FACSCanto II cytometer with a FSC threshold set at 10.000 and data analysis was conducted with BD FACSDiva software. A minimum of 40.000 of nucleated cells were acquired per sample. The following cell populations were detected: T lymphocytes ($CD11b^{-}CD3^{+}$), helper T cells (CD11b-CD3+CD8-CD4+), cytotoxic T cells ($CD11b^{-}CD3^{+}CD4^{-}CD8^{+}$), B lymphocytes ($CD11b^{-}CD3^{-}CD19^{+}$), neutrophils ($CD11b^{+}Ly6C^{-}Ly6G^{+}$), inflammatory monocytes ($CD11b^{+}Ly6G^{-}Ly6C^{high}$) and non-inflammatory monocytes ($CD11b^{+}Ly6G^{-}Ly6C^{low}$)[26]. The gating strategy is illustrated in ***Supplemental Figure 4***.

13. Western blot analysis in myocardial tissue

Western Blot analysis in myocardial tissue samples was performed as previously described[1,2,27]. At 2h or 24h of reperfusion (2nd and 5th series of experiments respectively), tissue samples from the ischemic myocardium were snap frozen in liquid nitrogen and subsequently pulverized in dry ice. The powders were homogenized using lysis solution (1% Triton X100, 20 mM Tris pH 7.4–7.6, 150mM NaCl, 50mM NaF,

1mM EDTA, 1mM ethylene glycol tetraacetic acid, 1mM glycerolphosphatase, 1% sodium dodecyl sulfate (SDS), 100mM phenylmethanesulfonyl fluoride, and 0.1% protease phosphatase inhibitor cocktail). Protein content was determined using Lowry method. An equal amount of protein was loaded in each well and then separated by SDS-polyacrylamide gel electrophoresis 8–17% and transferred onto a polyvinylidene difluoride membrane (PVDF). After blocking with 5% nonfat dry milk, membranes were incubated overnight at 4 °C with the following primary antibodies: ICAM-1 (dilution 1:1000, mouse mAb # MA5407, Invitrogen), MMP-2 (dilution 1:1000, Rabbit mAb #87809), TIMP-1 (dilution 1:1000, mouse mAb, sc-21734, Santa Cruz Biotechnology VEGF (dilution 1:1000, Mouse mAb, sc-7269, Santa Cruz Biotechnology), IGF -I (dilution 1:500, Rabbit mAb #73034), phospho-signal transducer and activator of transcription 3 (p-STAT3, Y705) (dilution 1:1000, Rabbit mAb #9145), t-STAT3 (dilution 1:1000, Mouse mAb #9139), fibroblast growth factor (FGF-2) (dilution 1:1000, mouse mAb, sc-74412, Santa Cruz Biotechnology), glyceraldehyde-3-phosphate dehydrogenase (GAPDH) (dilution 1:4000, Rabbit mAb #5174), beta-tubulin (dilution 1:2000, Rabbit mAb #2146) and α-actinin (dilution 1:3000, Rabbit mAb #6487). All antibodies were purchased from Cell Signaling Technology unless otherwise stated. Membranes were then incubated with secondary antibodies for 2 h at room temperature (Biorad (goat anti-mouse (#7076, dilution 1:2000) and goat anti-rabbit HRP (#7074, dilution 1:2000). Probed membrane signals were detected using chemiluminescent HRP substrate (Luminata Forte Western HRP substrate, Millipore, USA) and imaged using an automated cooled charge-coupled device imager (ImageQuant LAS 500, GE Healthcare Bio-Sciences, Sweden). Relative densitometry was determined using ImageJ 1.49v software (National Institutes of Health, USA) and the values for phospho-proteins and were normalized to the values for total respective proteins. A-actinin, β-tubulin and GAPDH were used as loading control.

14. Clinical study parameters

The baseline characteristics of patients are depicted in ***Supplemental Table 9***.

14.1. Sample size

The sample size was determined with power calculation a priori based on previously published values regarding the percent changes of perfused boundary region (ΔPBR%) after treatment with empagliflozin and insulin (-3.4% and -2.4%, respectively, with a SD = 1.2)[26]. To detect the ΔPBR% with $\alpha = 0.05$ and power = 0.80, at least 18 patients per treatment group are necessary for the conduction of clinical study.

14.2. Endothelial glycocalyx

The perfused boundary region (PBR) of the sublingual arterial microvessels with a diameter that ranged from 5 to 25μm was measured using Sidestream Dark Field imaging (Microscan, Glycocheck, Microvascular Health Solutions Inc, Salt Lake City, UT). This technique provides a fast and noninvasive assessment of the endothelial glycocalyx thickness[28]. The PBR is the cell-poor layer that results from the separation between the flowing red blood cell column and plasma on the surface of the vascular lumen. An increased PBR value indicates a deeper penetration of blood cells into the luminal part of the glycocalyx and is a precise marker of reduced glycocalyx thickness[28]. This technique was proposed as a valid technique to assess endothelial integrity by the European Society of Cardiology Working Group on Peripheral Circulation[29].

14.3. Flow-mediated dilation

Endothelium-dependent flow-mediated dilation (FMD) of the brachial artery was estimated in all subjects according to a previously published method[30]. All participants were studied in the morning, after abstinence from caffeine, alcohol, and food for 8 hours before the examination. All vasoactive medications were discontinued for 24 hours prior to the study. Optimal imaging of the right brachial artery was obtained and

a resting scan was recorded using ultrasonography (GE Logiq 7) and a 7.0-MHz transducer. Reactive hyperemia was induced by inflation of a cuff on the upper forearm to suprasystolic pressure for 5 minutes. The cuff was then rapidly deflated. The brachial artery was scanned continuously 30 seconds before and 90 seconds post-cuff deflation. All images were stored in a computerized station (EchoPac GE 202, Horten, Norway) and analyzed using off line software. Measurements of arterial diameters were obtained utilizing electronic calipers from the anterior to the posterior m-line. FMD was calculated as the percent increase in arterial diameter during hyperemia compared with the arterial diameter at rest. Inter- and intra-observer variability for repeated measurements of brachial artery diameter were 0.08 ± 0.19 mm and 0.1 ± 0.12 mm, respectively, in our laboratory.

14.4. Central hemodynamics and echocardiography

The carotid-femoral pulse wave velocity (PWV) was measured using tonometry by Complior (Alam Medical, Vincennes, France). Normal values of PWV were <10 m/s. Echocardiography studies were performed using a Vivid E95 (GE Medical Systems, Horten, Norway) ultrasound system and were digitally stored in a computerized station (EchoPac GE 202, Horten, Norway). All studies were analyzed by 2 observers (G.P. and D.B.), blinded to clinical and laboratory data.

14.5. Two-dimensional strain analysis

We measured LV global longitudinal strain (GLS;%) from 2-dimensional echocardiography images obtained with a frame rate of 70/s to 80/s, from the apical 4, 2-, and 3-chamber views using the 17 LV segment model and a dedicated software (EchoPac PC 203; GE Healthcare, Horten, Norway)[28]. The intraobserver and interobserver reproducibility values for LV strain and strain rate parameters were 8% and 9%, respectively.

14.6. ELISA assays

Patient's plasma was collected in heparin. The Human Thrombomodulin Elisa Kit (#Cat number EA100438, Origene) (https://www.origene.com/catalog/assay-kits/ELISA-kits/ea100576/human-thrombomodulin-ELISA-kit) was used and the samples were assessed undiluted or in a dilution 1:1 with sample diluent provided by the kit so that the measured values are in the range of the standard curve. The sensitivity of the kit is <10pg/ml. Also, the Human ICAM-1Elisa kit (#Cat number EA100244, Origene); (https://www.origene.com/catalog/assay-kits/elisa-kits/ea100244/human-icam-1-elisa-kit) was used and the samples were diluted 1:10 or 1:15 with sample diluent provided by the kit so that the measured values are in the range of the standard curve. The sensitivity of the kit is <10pg/ml. The procedure was performed according to the manufacturer's instructions. The ELISA plate output was measured on the microplate reader Tecan Infinite 200 PRO series at 450nm. The content of the samples in the respective markers was calculated based on the standard curves with a four-parameter logistic (4PL) curve fit upon correction for the background (on GraphPad Prism software). Dilution factors were multiplied by the output of each sample after 4PL analysis. The ELISA assays were performed by a blinded investigator (P.E.N.).

Supplemental Results

EMPA-Pre-AMI and EMPA-Post-AMI treatment significantly reduced the infiltration of all inflammatory cells in the heart (***Figures 4D and 4E).*** No differences were observed in T and B cells or the CD4/CD8 T cell ratio in the heart (***Supplemental Figure 9A***). In the peripheral blood, a decrease in LyC6^{low} monocytes was observed in Control-AMI and EMPA-Post-AMI in comparison to the Sham group (***Supplemental Figure 9B)*** and an increased recruitment of neutrophils was obvious in the EMPA-Post-AMI group in the spleen ***(Supplemental Figure 9C).***

Supplemental Table 1: Perfusion Buffer ingredients.

Reagent	Final concentration (mM)	Amount (g or as otherwise stated) for 1 liter
NaCl	120.4	7.03
KCl	14.7	1.1
KH_2PO_4	0.6	0.082
Na_2HPO_4	0.6	0.085
$MgSO_4$-7H_2O	1.2	0.30
Na-HEPES 1M solution	10	10 mL
$NaHCO_3$	4.6	0.39
Taurine	30	3.75
BDM	10	1
Glucose	5.5	1
H_2O		q.s. 1L

pH was adjusted to 7.0 with HCl as needed and the buffer was filter sterilized through a 0.2μm mesh.

Supplemental Table 2: Myocyte Digestion Buffer ingredients.

Reagent	Final concentration	Amount
Perfusion Buffer	-	50 mL
Collagenase II	2.4 mg/mL	120 mg

Prepared fresh right before use, to prevent inactivation of the enzyme.

Supplemental Table 3: Myocyte Stopping Buffer ingredients.

Reagent	Final concentration	Volume
Perfusion Buffer		18 mL
Fetal Bovine Serum (FBS)	10%	2 mL
100 mM $CaCl_2$	12.5 μM	2.5 μL

Supplemental Table 4: List of Primers used for real-time PCR and PCR protocols.

Gene (Mus Musculus)	Primer (5'-3') Forward	Primer (5'-3') Reverse
Gapdh	CCCAGCTTAGGTTCATCAGGT	GCCAAATCCGTTCACACCG
Mmp-2	CAGTGATGGCTTCCTCTGGT	GTAAACAAGGCTTCATGGGGG
Mmp-14	CCACAAAGATGCCCCCTCAA	TTCCCGTCACAGATGTTGGG
Mmp-23	TGCCCCAGAAGCTGTGATTT	TCGTTCTGGTGGGTGATGTG
Timp-1	GGACCTGGTCATAAGGGCTAAAT	ATTTCCCACAGCCTTGAATCCT
Timp-2	CTTGCTACATCTCCTCCCCG	TGGTGCCCATTGATGCTCTT
Igf-1	ACCTCAGACAGGCATTGTGG	GTGGGGCACAGTACATCTCC
Egfr	CACTGCTGGTGTTGCTGAC	TGTGCCTTGGCAGACTTTCT
Vegfa	CTGGACCCTGGCTTTACTGC	ACTTGATCACTTCATGGGACTTCT
Vegfb	AGCCAGACAGGGTTGCCAT	TGGATGATGTCAGCTGGGGAG
Syndecan-2	TGCCATTTTCCTCATCCTGCT	TGGTTTGCGTTCTCCAAGGT
Sod-3	TTCTACGGCTTGCTACTGGC	GGTCAAGCCTGTCTGCTAGG
Rab7b	GAGGGATGATGTCCTGGCAA	CGATTTTGTTCCCCAGCACC
Icam	AGTCCGCTGTGCTTTGAGAA	CTCTCCGGAAACGAATACACG

Supplemental Table 5: Concentration of EMPA in murine plasma

Data are presented as mean ± SD.

Timepoint	Concentration of EMPA (ng/mL)
**1st dose**	**412.0±75.5**
**2nd dose**	**398.3± 64.1**
**3rd dose**	**408.0± 180.4**

Supplemental Table 6: No-reflow and infarct size results.

Data are presented as mean ± SD.

	Sham (n=8)	Control-AMI (n=13)	EMPA-Pre-AMI (n=14)	EMPA-Post-AMI (n=11-13)
**No-reflow to area at risk**		21.3±12	4.5±2.8	6.3±3.9
**No-reflow to left ventricle**		14.3±8.9	2.9±2.0	4.2±2.6
**No-reflow to infarct size**		44.6 ±16.9	24.4±13.7	33.6±14.3
**Infarct size to area at risk (TTC Staining)**		44.4±13.3	17.8±5.5	26.6±4.2
**Infarct size to left ventricle (TTC staining)**	6.7±3.8	29.8±10.9	11.4±3.3	18.56±11.9
**Infarct size to left ventricle (CMR)**	5.4±3.0	28.4±17	8.9±6.4	14.3±3.3

Supplemental Table 7: Cardiac magnetic resonance scanning parameters.

	CINE SAX	CINE 2CH/3CH/4CH	LGE SAX	LGE 2CH/3CH/4CH
**Number of slices (n)**	7	1	7	1
**Image resolution (pixels)**	167 x 167	167 x 167	256 x 256	256 x 256
**Field of view**	25 x 25	25 x 25	25 x 25	25 x 25
**Repetition time (ms)**	5.1*	5.1*	36*	36*
**Echo time (ms)**	1.1	1.1	1.1	1.1
**Flip angle (°)**	22	22	65	65
**Slice thickness (um)**	800	800	800	800
**Phases**	30	1	1	1

**Small variations due to ECG triggering.*

2CH, 2 chamber long axis; 3CH, 3 chamber long axis; 4CH, 4 chamber long axis; LGE, late gadolinium enhancement; n, number; SAX, short axis.

Supplemental Table 8: Cardiac magnetic resonance analysis results.

Data are presented as mean ± SD.%GLS: % global longitudinal strain; %GCS: %global circumferential strain.

	**Sham (n=8)**	**Control-AMI (n=13)**	**EMPA-Pre-AMI (n=14)**	**EMPA-Post-AMI (n=13)**
**End diastolic volume (μL)**	66.78±3.2	72.87±3.2	67.77±1.6	37.40±3.0
**End systolic volume (μL)**	28.97±1.8	42.79±4.8	28.64±2.1	32.82±3.8
**Ejection fraction (%)**	57.11±1.6	42.70±4.3	58.14±2.4	52.67±3.7
**Cardiac output (mL/min)**	17.15±0.9	13.54±1.2	17.61±0.6	15.56±0.9
**Stroke volume (mL/min)**	38.11±2.0	30.08±2.6	39.13±1.4	34.58±1.9
**Cardiac mass (mg)**	53.11±2.7	61.53±3.4	53.11±1.6	56.14±1.8
**GCS (%)**	-34.52±1.1	-23.34±3.0	-34.72±1.8	-30.06±2.7
**GLS (%)**	-18.22±1.0	-12.177±1.4	-18.50±0.8	-17.21±1.4

Supplemental Table 9: Baseline patients' characteristics.

Data are presented as mean ± SD, number (percentage), or median (first quartile – third quartile). Continuous variables were compared with the Student t test. Binary variables were compared with the chi-squared test. BMI, body mass index; LVEF, left ventricular ejection fraction; HbA1c, glycosylated hemoglobin; eGFR, estimated glomerular filtration rate; CAD, coronary artery disease; ACEi, angiotensin-converting enzyme inhibitors; ARB, angiotensin receptor blockers; CCB, calcium channel blockers; MRA, mineralocorticoid receptor antagonists.

	**Empagliflozin** (n = 24)	**Control** (n = 18)	**P-value**
**Age, years**	58 ± 8	59 ± 7	0.52
**Male sex, n (%)**	22 (92)	16 (89)	0.76
**Duration of diabetes, years (range)**	7 (2 – 10)	8 (2 – 12)	0.34
**BMI, Kg/m^2**	29.3 ± 4.0	29.8 ± 5.0	0.71
**LVEF, %**	53.8 ± 6.9	53.5 ± 5.5	0.92
**HbA1c, %**	8.0 ± 0.9	8.2 ± 1.2	0.66
**Creatinine, mg/dL**	1.1 ± 0.2	1.0 ± 0.3	0.85
**eGFR, mL/min per 1.73 m^2**	85 ± 10	86 ± 9	0.74
**Risk factors, n (%)**			
**Hypertension**	15 (62.5)	9 (50)	0.42
**Dyslipidemia**	24 (100)	18 (100)	>0.99
**Current smoking**	12 (50)	7 (39)	0.47
**Family history of CAD**	11 (46)	9 (50)	0.79
**Cardiovascular medications, n (%)**			
**ACEi/ARB**	24 (100)	18 (100)	>0.99
**CCB**	7 (29)	4 (22)	0.61
**β-Blockers**	24 (100)	18 (100)	>0.99

**Diuretics**	5 (21)	6 (33)	0.21
**MRA**	1 (4)	3 (17)	0.17
**Statins**	24 (100)	18 (100)	>0.99
**Fibrates**	3 (12.5)	1 (5.5)	0.45
**Antiplatelets**	24 (100)	18 (100)	>0.99

.

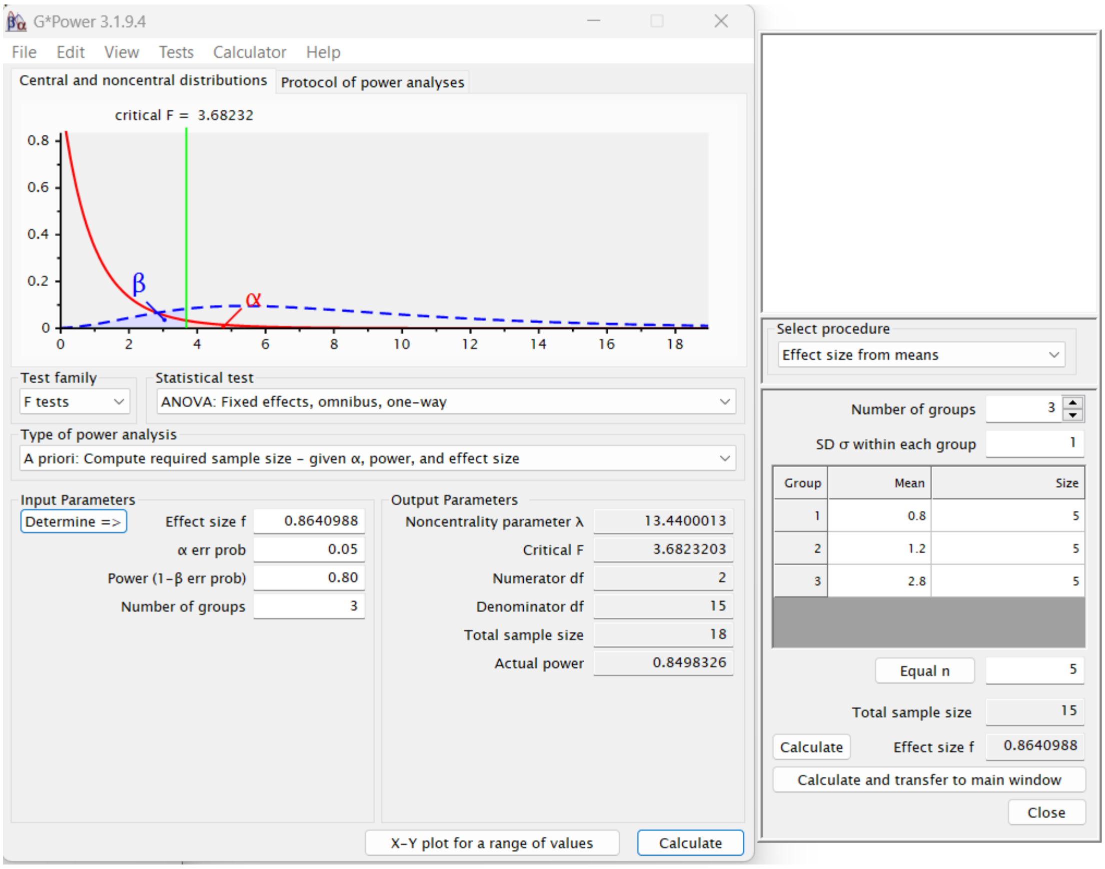

Supplemental Figure 1: A priori G power analysis for sample size estimation of the second series of experiments. *Snapshot of G Power analysis software depicting the results of the analysis. The sample size of our study regarding the mRNA expression calculation is n=18 animals, n=6 per group.*

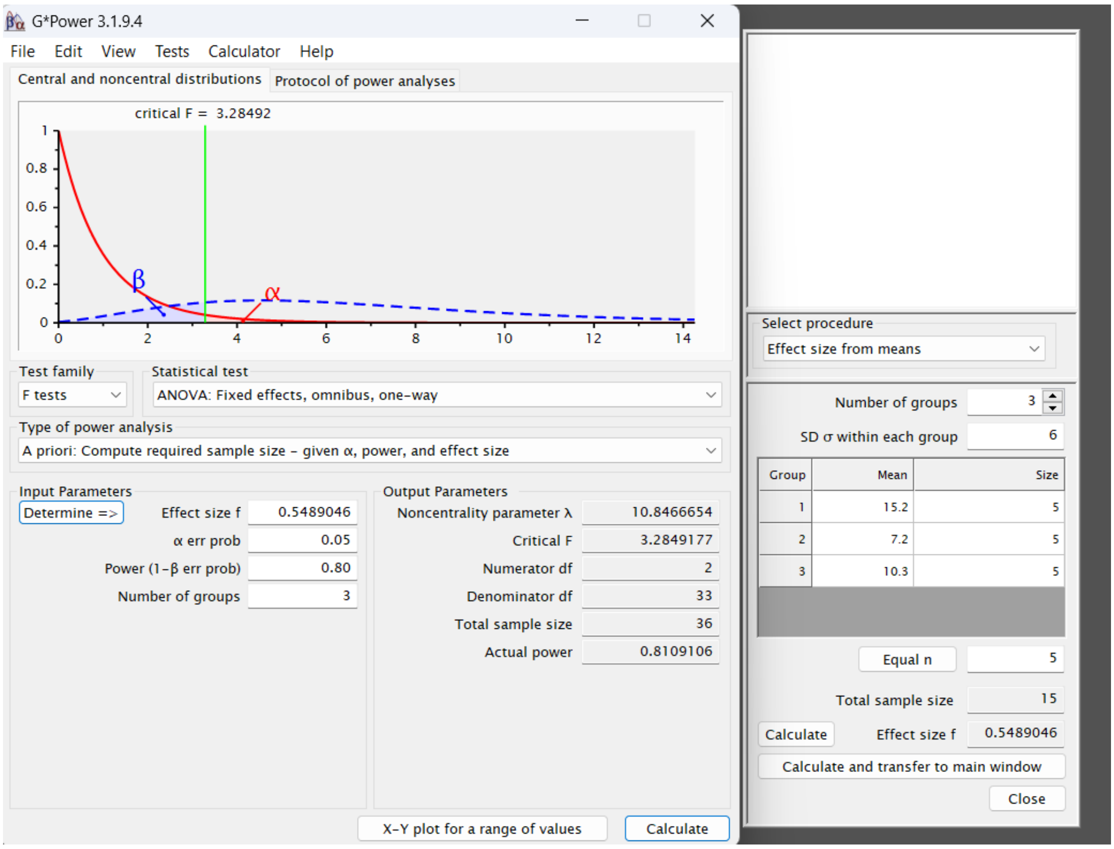

Supplemental Figure 2: A priori G power analysis for sample size estimation of the fourth series of experiments. *Snapshot of G Power analysis software depicting the results of the analysis. The sample size of our study regarding the primary outcome "no reflow" calculation is n=36 animals, n=12 per group.*

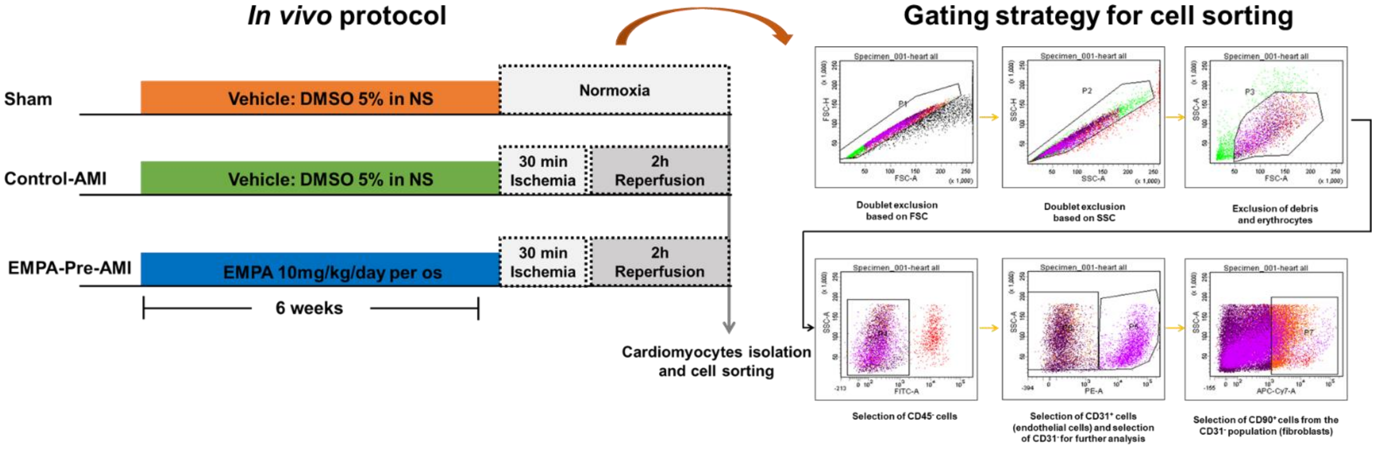

Supplemental Figure 3: Schematic representation of the experimental protocol to examine the effect of AMI and empagliflozin on the transcriptome of cardiomyocytes, fibroblasts and ECs at 2 h after reperfusion. *On the left panel, a schematic representation of the in vivo protocol including the interventions to the animals which included 6 weeks administration of vehicle or empagliflozin prior to 30 min of ischemia and 2 h after reperfusion is depicted. On the right panel, the gating strategy for the cell sorting is presented. The FSC-H versus FSC-A and SSC-H versus SSC-A plots were used for the exclusion of doublets while the P3 gate was used to exclude debris. P4 gate demonstrates the CD45$^-$ population, gate P5 are CD45$^-$ and CD31$^+$ events (ECs) and the P7 gate includes the CD45$^-$, CD31$^+$ and CD90$^+$ events (FBs). ECs: endothelial cells; FSC: Forward scatter; SSC: side scatter.*

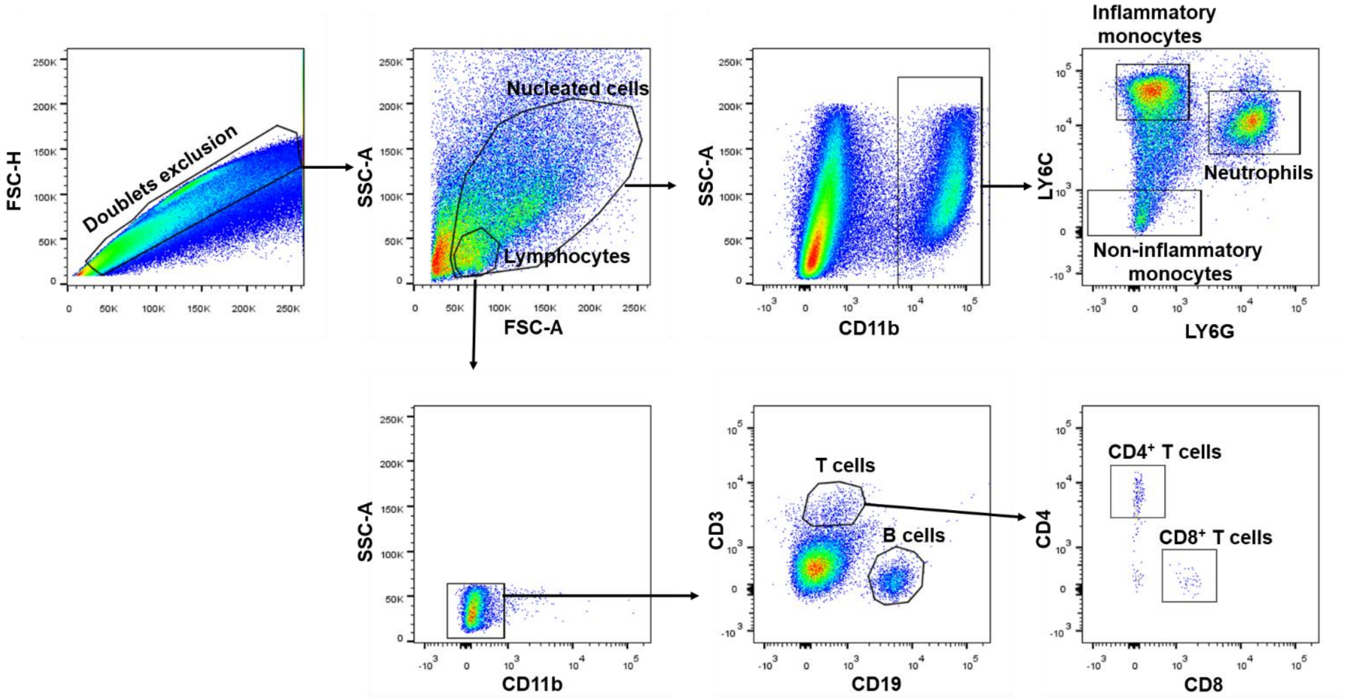

Supplemental Figure 4: General gating strategy for the evaluation of immune subsets in the myocardium. *Total events analyzed were first gated for debris and doublets exclusion and were then plotted on a SSC/CD11b dot plot. CD11b^{+} cells were further analyzed on a Ly6G/Ly6C dot plot for the discrimination of monocytes and neutrophils, whereas the CD11b^{-} lymphocytes on a CD19/CD3 dot plot for the discrimination of B cells and T cells respectively. T cells were further analyzed on a CD4/CD8 dot plot for the discrimination of CD4^{+} and CD8^{+} T cells.*

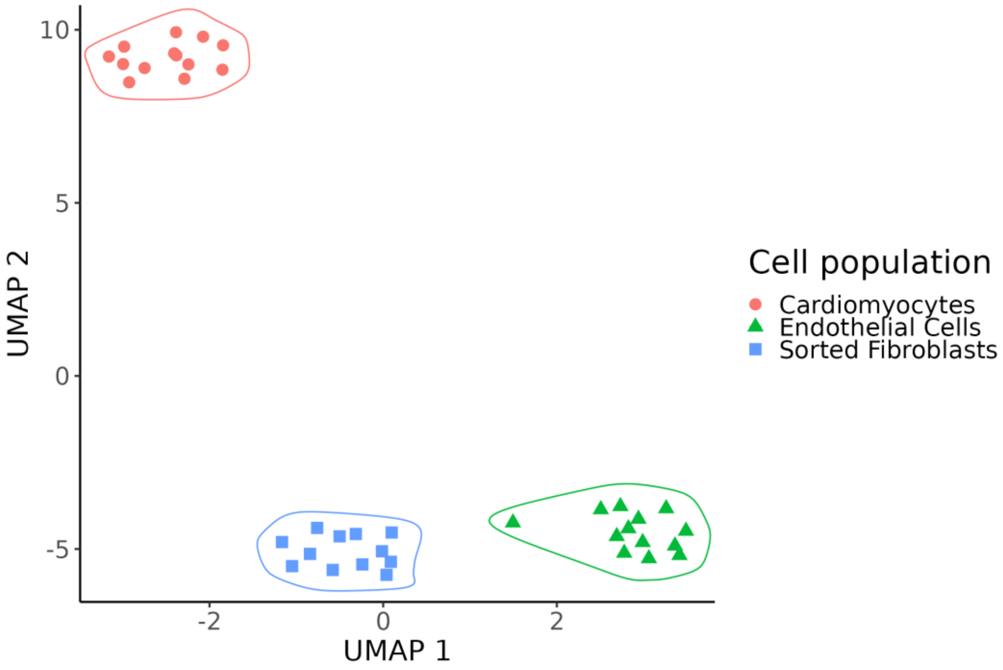

***Supplemental Figure 5**: **Discrimination of the cardiac cell populations based on their transcriptome**. UMAP plot using the PanglaoDB database and a decomposition package to demonstrate the effective discrimination of the cell populations' transcriptome.*

A

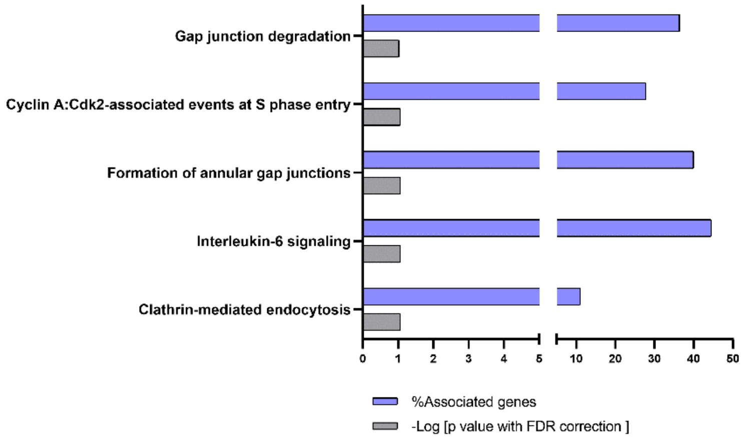

B

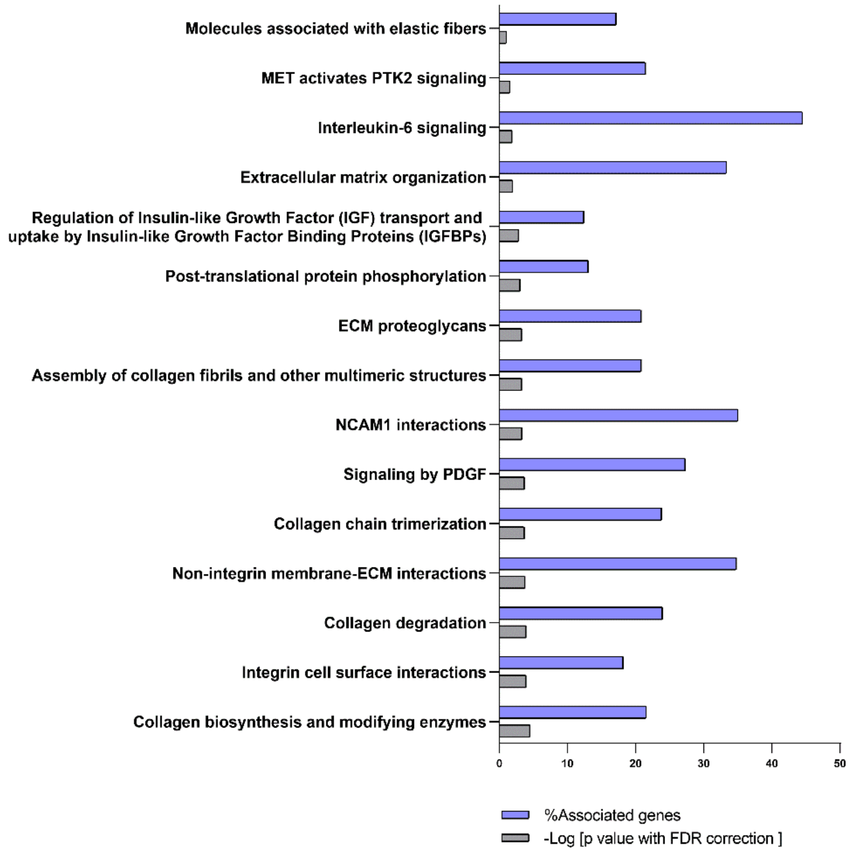

***Supplemental Figure 6**: **Pathway enrichment analysis in fibroblasts and endothelial cells for the comparison between Sham and Control-AMI groups**. Bar plots indicating the significant Reactome pathways between Sham and Control-AMI groups at 2 h after reperfusion in A) sorted fibroblasts with FDR<0.1 and B) sorted ECs with FDR<0.05. ECs: endothelial cells*

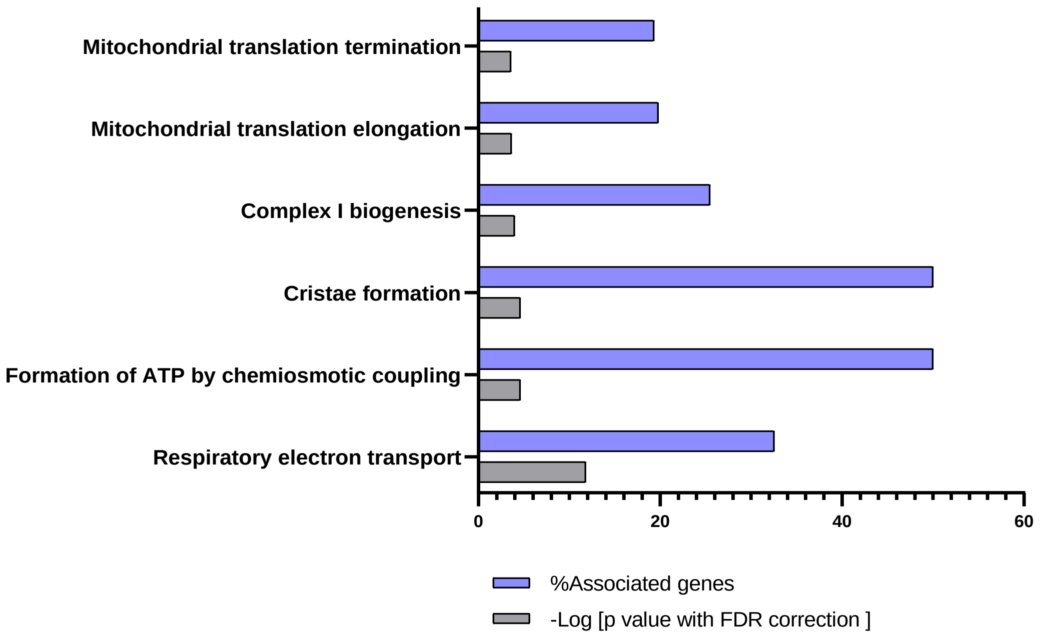

***Supplemental Figure 7**: **Pathway enrichment analysis in cardiomyocytes for the comparison between Control-AMI and EMPA-Pre-AMI groups**. Bar plots indicating the significant Reactome pathways between Control-AMI and EMPA-Pre-AMI groups at 2 h after reperfusion in cardiomyocytes with FDR<0.05. Significant genes with FDR<0.1 were used for pathway analysis.*

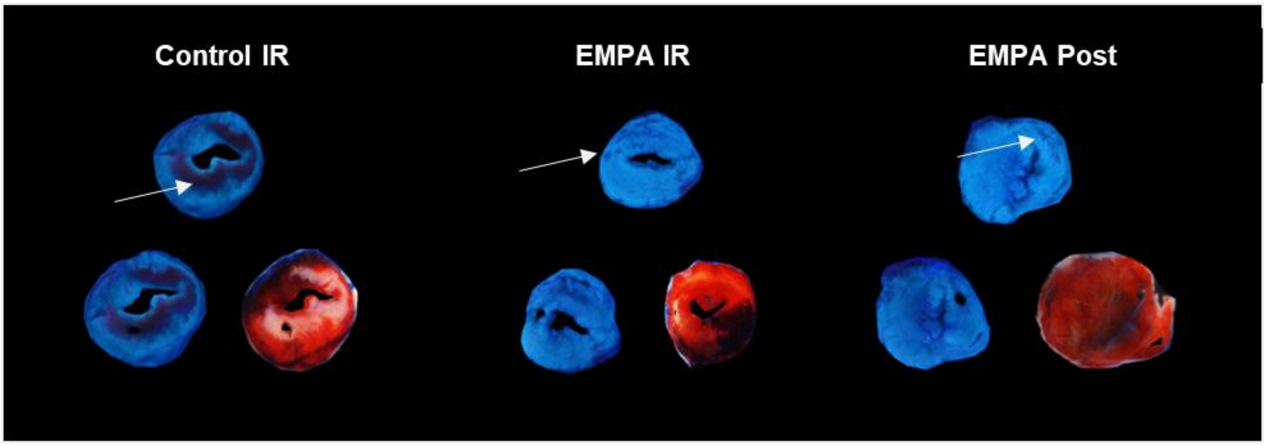

Supplemental Figure 8: Representative images of stained heart slices with Thioflavin S and TTC staining at 48 h after reperfusion. *Biopsies were obtained from the center of the infarcted no-reflow area per group for transition electron microscopy. The spot was chosen after TTC staining to ensure the location of the biopsy area in the infarcted core and under UV light to ensure the biopsy was derived from a thioflavin S negative area as depicted in the upper panel (arrows). A verification picture was obtained after the biopsy was removed.*

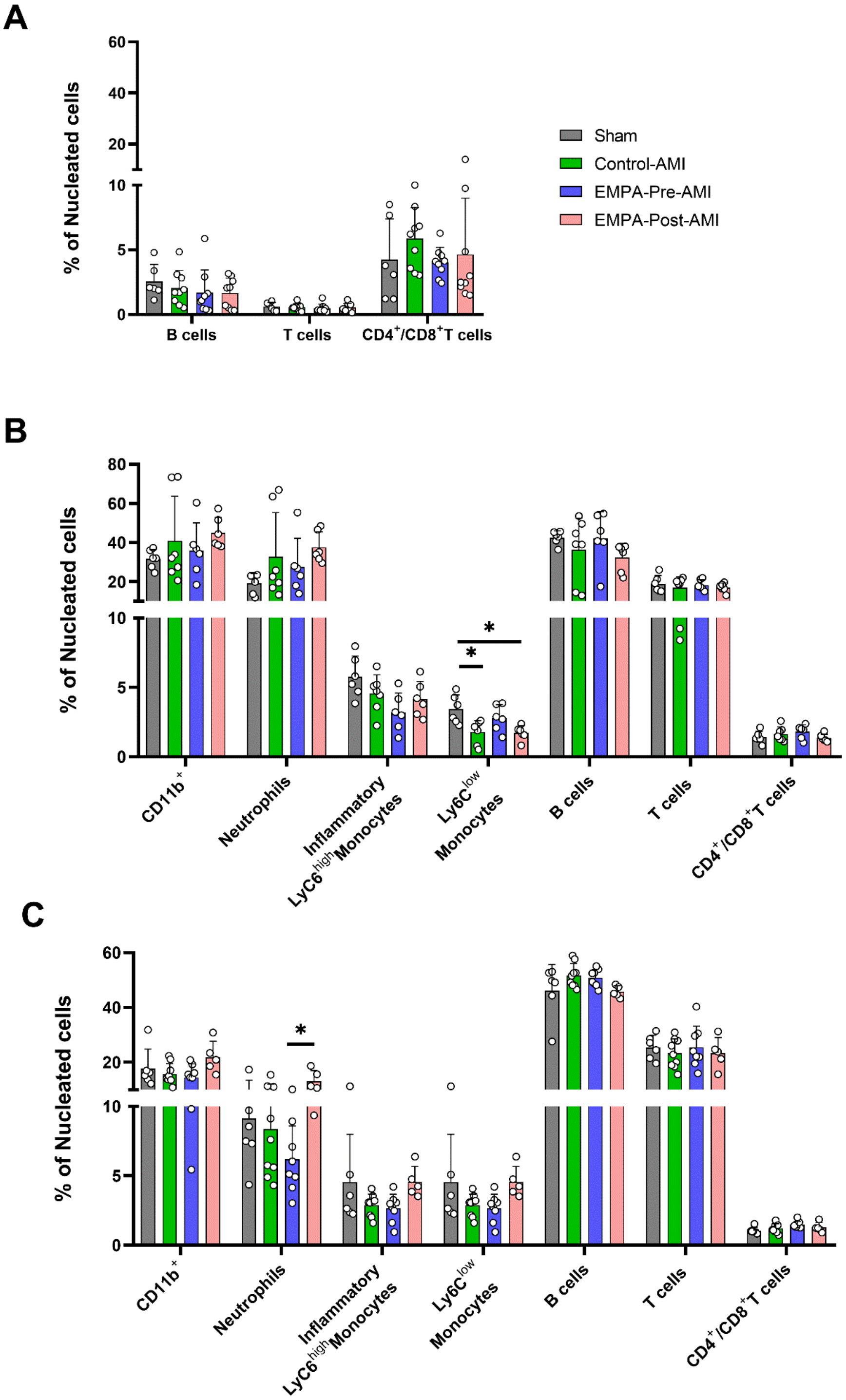

Supplemental Figure 9: Dot plots with % of various cell populations to nucleated cells in the A) ischemic part of the myocardium, B) peripheral blood and C) the spleen. *In the peripheral blood, a decrease in LyC6^{low} monocytes was observed in Control-AMI and EMPA-Post-AMI in comparison to the Sham group and an increased recruitment of neutrophils was obvious in the EMPA-Post-AMI group* in the spleen *(n=6 for the Sham group and n=9 per group for Contro-AMI, EMPA-Pre-AMI and EMPA-Post-AMI in the heart and n=6 per group for the peripheral blood and the spleen). Bars indicate means ± SD. One-way ANOVA with Tukey's as post-Hoc analysis was employed for the comparisons among the groups (*p<0.05.).*

References

1. Nikolaou PE, Efentakis P, Abu Qourah F, et al. Chronic Empagliflozin Treatment Reduces Myocardial Infarct Size in Nondiabetic Mice Through STAT-3-Mediated Protection on Microvascular Endothelial Cells and Reduction of Oxidative Stress. *Antioxid Redox Signal*. 2021;34:551–571.

2. Nikolaou PE, Mylonas N, Makridakis M, et al. Cardioprotection by selective SGLT-2 inhibitors in a non-diabetic mouse model of myocardial ischemia/reperfusion injury: a class or a drug effect? *Basic Res Cardiol*. 2022;117:27.

3. Andreadou I, Efentakis P, Balafas E, et al. Empagliflozin Limits Myocardial Infarction in Vivo and Cell Death in Vitro: Role of STAT3, Mitochondria, and Redox Aspects. *Frontiers in Physiology*. 2017;8.

4. Bøtker HE, Hausenloy D, Andreadou I, et al. Practical guidelines for rigor and reproducibility in preclinical and clinical studies on cardioprotection. *Basic Res Cardiol*. 2018;113:39.

5. Schurch NJ, Schofield P, Gierliński M, et al. How many biological replicates are needed in an RNA-seq experiment and which differential expression tool should you use? *RNA*. 2016;22:839–851.

6. Tahara A, Takasu T, Yokono M, Imamura M, Kurosaki E. Characterization and comparison of sodium-glucose cotransporter 2 inhibitors in pharmacokinetics, pharmacodynamics, and pharmacologic effects. *Journal of Pharmacological Sciences*. 2016;130:159–169.

7. Santos-Gallego CG, Requena-Ibáñez JA, Picatoste B, et al. Cardioprotective Effect of Empagliflozin and Circulating Ketone Bodies During Acute Myocardial Infarction. *Circ Cardiovasc Imaging*. 2023;16:e015298.

8. Konijnenberg LSF, Luiken TTJ, Veltien A, et al. Imatinib attenuates reperfusion injury in a rat model of acute myocardial infarction. *Basic Res Cardiol*. 2023;118:2.

9. Tsoumani M, Georgoulis A, Nikolaou P-E, et al. Acute administration of the olive constituent, oleuropein, combined with ischemic postconditioning increases myocardial protection by modulating oxidative defense. *Free Radic Biol Med*. 2021;166:18–32.

10. Rusinkevich V, Huang Y, Chen Z-Y, et al. Temporal dynamics of immune response following prolonged myocardial ischemia/reperfusion with and without cyclosporine A. *Acta Pharmacol Sin*. 2019;40:1168–1183.

11. O'Connell TD, Rodrigo MC, Simpson PC. Isolation and Culture of Adult Mouse Cardiac Myocytes. In: Vivanco F, editor. *Cardiovascular Proteomics: Methods and Protocols*. Methods in Molecular Biology™. Totowa, NJ: Humana Press, 2007:271–296.

12. Efentakis P, Varela A, Chavdoula E, et al. Levosimendan prevents doxorubicin-induced cardiotoxicity in time- and dose-dependent manner: implications for inotropy. *Cardiovasc Res*. 2020;116:576–591.

13. Basu C, Cannon PL, Awgulewitsch CP, Galindo CL, Gamazon ER, Hatzopoulos AK. Transcriptome analysis of cardiac endothelial cells after myocardial infarction reveals temporal changes and long-term deficits. *Sci Rep*. 2024;14:9991.

14. Martin M. Cutadapt removes adapter sequences from high-throughput sequencing reads. *EMBnet.journal*. 2011;17:10–12.

15. Patro R, Duggal G, Love MI, Irizarry RA, Kingsford C. Salmon provides fast and bias-aware quantification of transcript expression. *Nat Methods*. 2017;14:417–419.

16. Martin FJ, Amode MR, Aneja A, et al. Ensembl 2023. *Nucleic Acids Research*. 2023;51:D933–D941.

17. Jew B, Alvarez M, Rahmani E, et al. Accurate estimation of cell composition in bulk expression through robust integration of single-cell information. *Nat Commun*. 2020;11:1971.

18. Franzén O, Gan L-M, Björkegren JLM. PanglaoDB: a web server for exploration of mouse and human single-cell RNA sequencing data. *Database*. 2019;2019:baz046.

19. Robinson MD, McCarthy DJ, Smyth GK. edgeR: a Bioconductor package for differential expression analysis of digital gene expression data. *Bioinformatics*. 2010;26:139–140.

20. Robinson MD, Oshlack A. A scaling normalization method for differential expression analysis of RNA-seq data. *Genome Biology*. 2010;11:R25.

21. Benjamini Y, Hochberg Y. Controlling the False Discovery Rate: A Practical and Powerful Approach to Multiple Testing. *Journal of the Royal Statistical Society: Series B (Methodological)*. 1995;57:289–300.

22. Gillespie M, Jassal B, Stephan R, et al. The reactome pathway knowledgebase 2022. *Nucleic Acids Research*. 2022;50:D687–D692.

23. Blighe K. PCAtools: everything Principal Component Analysis. 2023.

24. Wickham H. *ggplot2*. Cham: Springer International Publishing, 2016.

25. Arslan F, Lai RC, Smeets MB, et al. Mesenchymal stem cell-derived exosomes increase ATP levels, decrease oxidative stress and activate PI3K/Akt pathway to enhance myocardial viability and prevent adverse remodeling after myocardial ischemia/reperfusion injury. *Stem Cell Research*. 2013;10:301–312.

26. Efentakis P, Choustoulaki A, Kwiatkowski G, et al. Early microvascular coronary endothelial dysfunction precedes pembrolizumab-induced cardiotoxicity. Preventive role of high dose of atorvastatin. *Basic Res Cardiol*. 2024. Published onlineMarch 23, 2024. https://doi.org/10.1007/s00395-024-01046-0.

27. Nikolaou P-E, Boengler K, Efentakis P, et al. Investigating and re-evaluating the role of glycogen synthase kinase 3 beta kinase as a molecular target for cardioprotection by using novel pharmacological inhibitors. *Cardiovasc Res*. 2019;115:1228–1243.

28. Ikonomidis I, Pavlidis G, Thymis J, et al. Effects of Glucagon-Like Peptide-1 Receptor Agonists, Sodium-Glucose Cotransporter-2 Inhibitors, and Their Combination on Endothelial Glycocalyx, Arterial Function, and Myocardial Work Index in Patients With Type 2 Diabetes Mellitus After 12-Month Treatment. *J Am Heart Assoc*. 2020;9:e015716.

29. Lekakis J, Abraham P, Balbarini A, et al. Methods for evaluating endothelial function: a position statement from the European Society of Cardiology Working Group on Peripheral Circulation. *Eur J Cardiovasc Prev Rehabil*. 2011;18:775–789.

30. Ikonomidis I, Lekakis JP, Nikolaou M, et al. Inhibition of interleukin-1 by anakinra improves vascular and left ventricular function in patients with rheumatoid arthritis. *Circulation*. 2008;117:2662–2669.
